# 3D structure and stability prediction of DNA with multi-way junctions in ionic solutions

**DOI:** 10.1371/journal.pcbi.1013346

**Published:** 2025-08-18

**Authors:** Xunxun Wang, Ya-Zhou Shi

**Affiliations:** 1 Guizhou Key Laboratory of Microbio and Infectious Disease Prevention & Control, School of Biology and Engineering, Guizhou Medical University, Guiyang, China; 2 Research Center of Nonlinear Science, School of Mathematics & Statistics, Wuhan Textile University, Wuhan, China; KU: The University of Kansas, UNITED STATES OF AMERICA

## Abstract

Understanding the three-dimensional (3D) structure and stability of DNA is essential for elucidating its biological functions and advancing structure-based drug design. Here, we present an improved coarse-grained (CG) model for *ab initio* prediction of DNA folding, integrating a refined electrostatic potential, replica-exchange Monte Carlo simulations, and weighted histogram analysis. The model accurately predicts the 3D structures of DNA with multi-way junctions (e.g., achieving a mean RMSD of ~8.8 Å for top-ranked structures across four DNAs with three- or four-way junctions) from sequence, outperforming existing fragment-assembly and AI-based approaches. The model also reproduces the thermal stability of junctions across diverse sequences and lengths, with predicted melting temperatures deviating by less than 5 °C from experimental values, under both monovalent (Na⁺) and divalent (Mg^2^⁺) ionic conditions. Furthermore, analysis of the thermal unfolding pathways reveals that the overall stability of multi-way junctions is primarily determined by the relative free energies of key intermediate states. These results provide a robust framework for predicting complex DNA architectures and offer mechanistic insights into DNA folding and function.

## Author summary

Beyond the familiar double helix, DNA can fold into complex three-dimensional (3D) structures such as multi-way junctions that are essential in biological processes and widely used in nanotechnology and drug design. Predicting how these structures form and how stable they are, especially under salt conditions, remains a major challenge. In this study, we introduce a coarse-grained model that can accurately predict both the 3D structures and thermal stability of three- and four-way DNA junctions directly from their sequence. By integrating a refined treatment of electrostatic interactions and advanced sampling techniques (e.g., REMC and WHAM), the model reproduces experimental 3D structures and melting temperatures across a wide range of sequences and salt conditions including monovalent (Na⁺) and divalent (Mg^2^⁺) ions. We also find that the stability of these DNA junctions is governed by the relative energies of intermediate folding states. This work provides a practical tool for researchers to model complex DNA architectures and deepens our understanding of how DNA sequence and ions together control DNA structure and function.

## Introduction

DNA is a fundamental macromolecule in living organisms, essential for the storage and transmission of genetic information [1], regulation of protein synthesis [2], and control of gene expression [3]. Beyond its classical B-form double helix, DNA can adopt a variety of complex 3D structures, such as hairpins, junctions, quadruplexes, and other non-canonical forms, which are increasingly recognized as functional elements involved in gene expression regulation, genome stability, and epigenetic control [4–7]. These dynamic conformations are not only critical for biological processes, e.g., facilitating enhancer-promoter communication or transcription factor binding through structure folding, but also form the structural basis for DNA-based nanotechnology [8]. Among them, DNA with multi-way junctions represents a higher level of structural complexity compared to linear or duplex DNA [9], and is thought to contribute to molecular recognition, signaling, and the assembly of nucleic acid-based nanodevices [10]. Therefore, gaining deeper insights into the 3D structure and stability of such junction-containing DNAs is essential for both understanding their biological relevance and advancing DNA-based material design.

The DNA 3D structures can be experimentally resolved through experimental techniques such as X-ray crystallography, nuclear magnetic resonance spectroscopy, and cryo-electron microscopy [11,12]. However, due to the high cost and technical difficulty of these methods, the number of determined DNA 3D structures deposited in the Protein Data Bank (PDB) remains limited [11–14], especially in contrast to the vast number of DNA sequences available in GenBank [15,16]. To complement experimental approaches, several theoretical and computational methods have been developed to predict/model DNA 3D structures [14,17]. These methods can be classified into three categories: deep learning-based, template-based, and physics-based approaches.

Deep learning-based approaches have achieved remarkable success in protein structure prediction and are increasingly being applied to nucleic acids [18–22]. For example, AlphaFold3 [18,19] has recently demonstrated the ability to model nucleic acid structures by learning from large datasets encompassing proteins, RNA, and DNA. These models use neural network architectures to directly infer structural patterns from sequence data, enabling rapid and scalable predictions. However, their performance on diverse DNA/RNA topologies remains limited due to the relatively sparse and biased training data for nucleic acids (e.g., dominated by canonical double-helical structures), compared to the extensive and diverse datasets available for proteins [23,24]. On the contrary, template-based fragment assembly methods offer a flexible framework for constructing 3D structures of DNA with arbitrary topologies including multi-way junctions by assembling known structural fragments based on the secondary structure. A representative example is the 3dDNA, which extends the original 3dRNA platform to support DNA modeling [25,26]. It assembles DNA 3D structures with high accuracy by aligning input sequence and secondary structure information with a curated template library of experimentally determined structure segments [26]. However, these methods rely heavily on accurate secondary structure input, which remains challenging for DNAs with noncanonical or complex folds [27]. Additionally, the limited diversity of template libraries could restrict their effectiveness in de novo predictions for novel sequences.

Physics-based approaches can model DNA folding and structure formation by simulating fundamental physical interactions, without requiring structure templates or secondary structure information. All-atom molecular dynamics (MD) simulations using well-established force fields such as CHARMM [28,29] and AMBER [30–32] have been widely applied to study the microscopic behavior of dsDNA, including its dynamics, flexibility, and structure transitions. However, their high computational cost restricts these simulations to small DNA fragments and short time scales (falling short of capturing folding processes). To address these limitations, a variety of coarse-grained (CG) models have been developed by reducing the degrees of freedom while retaining essential physical and thermodynamic characteristics [33–43]. For example, the oxDNA model represents nucleotides as rigid bodies with backbone, stacking, and hydrogen bonding interactions, and has been widely used to simulate both single- and double-stranded DNA behavior (e.g., mechanics and thermodynamics), as well as large-scale DNA nanostructures such as origami and nano-tweezers [39]. The 3SPN model adopts a three-site representation (phosphate, sugar, base), incorporating base-pairing and stacking to capture DNA denaturation, persistence length, and local curvature [40]. Similarly, another three-interaction-site model of TIS-DNA uses sequence-specific stacking energies derived from dimer thermodynamics, enabling accurate modeling of force-extension behavior, elasticity, and melting temperatures of simple DNAs [41]. Despite their successes, these models typically rely on Gö-like potentials that encode native structural information, limiting their capacity for structure prediction directly from sequence. To overcome this, several physics-based CG models have been proposed to fold DNAs without using the predefined structure restraints. For instance, the NARES-2P, with two CG beads for one nucleotide, can reproduce duplex formation, melting temperatures, and mechanical stability of dsDNAs just from two separate strands [42]. The HiRE-RNA, using six to seven beads per nucleotide, enables the study of both structural dynamics and sequence-dependent melting profiles [43]. However, the parameters of these models may need further validation for quantifying thermodynamic and 3D structure to accord with experiments, especially for ssDNA.

In addition, DNA folding and dynamics are further influenced by its polyanionic nature, making ionic conditions (e.g., Na^+^ and Mg^2+^) crucial for accurate structural modeling [44–48]. Several existing CG models, including 3SPN, oxDNA, TIS-DNA, NARES-2P, and HiRE-RNA, incorporate electrostatic interactions using the Debye-Hückel approximation or mean-field multipole–multipole potentials to reproduce monovalent salt-dependent structural properties of DNA, such as persistence length, torsional stiffness or melting temperatures [49–53]. However, these models often neglect the significant influence of divalent ions on DNA/RNA folding. Recent studies have shown that explicitly including divalent cations, like Mg^2+^, enhances the accuracy of ion-nucleic acid and ion-ion interaction predictions, providing a more precise representation of the complex ionic environment around nucleic acids and better insights into the divalent ion effects on folding thermodynamics [54,55]. Very recently, we have also proposed a three-bead CG model to predict 3D structure folding for DNAs in monovalent/divalent ion solutions from sequence [13]. By integrating sequence-dependent base-pairing, base-stacking, and coaxial stacking interactions, along with an implicit electrostatic potential, the model accurately folded 20 dsDNAs (≤52 nt) and 20 ssDNAs (≤74 nt) into native-like structures (mean RMSD < 4Å), and quantitatively predicted melting temperatures (mean deviation < 3.0 °C) for 27 dsDNAs (including those with bulge loops and internal loops) and 24 ssDNAs (including hairpins and a pseudoknot). Despite these advances, accurately predicting the 3D folding of complex DNAs with multi-way junctions directly from sequence, especially under ionic conditions, remains a significant challenge for current models, including ours.

In this work, we have further refined our previously developed CG model by incorporating a structure-based electrostatic potential, an improved sampling algorithm (Replica Exchange Monte Carlo, REMC), and with the weighted histogram analysis method (WHAM). Specifically, the updated model includes: (i) a refined energy term to account for electrostatic energy term to capture electrostatic interactions between DNA and monovalent/divalent ions; (ii) the use of a more efficient REMC to enhance conformational sampling efficiency compared to conventional simulated annealing [13,56,57]; (iii) WHAM to analyze the thermal stability of DNA; and (iv) an all-atom reconstruction algorithm to recover atomistic structures from CG predictions. We applied the model to predict the 3D structures and thermodynamic stability of DNA with multi-way junctions in both monovalent and divalent ion solutions, and further analyzed their thermal unfolding pathways.

## Materials and methods

### Coarse-grained representation for DNA structures

**Fig 1:**
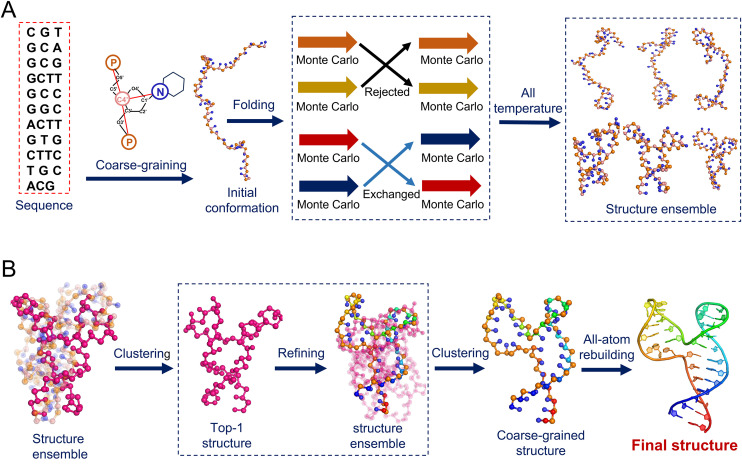
Schematic overview of the coarse-grained (CG) modeling framework for DNA folding. **(A)** The folding progress of DNA is based solely on sequence information, incorporating the coarse-grained representation of DNA, the initial conformation, REMC simulations, and a structure ensemble derived from ten different temperature replicas to explore conformational diversity. **(B)** Refinement of the DNA structure, beginning with the identification of the most probable conformation through clustering of low-energy conformations, followed by the reconstruction of an all-atom model.

In the present model, each DNA nucleotide is represented by a set of three CG beads [13]. Specifically, one bead is assigned to the phosphate group (P), another to the sugar moiety centered at the C4ʹ atom (C), and the third to the nucleobase (N), represented by the N1 atom in pyrimidines or the N9 atom in purines (Fig 1A). This streamlined model retains the essential structural and chemical properties of DNA, such as backbone connectivity and base-pairing interactions [55,57]. The three types of beads are modeled as spheres with van der Waals radii of 1.9 Å (P), 1.7 Å (C), and 2.2 Å (N), respectively, and the P bead carries a unit negative charge to account for the polyanionic nature of DNA.

### Coarse-grained force field

In the present model, the total energy of a DNA chain is given by:


$$U=U_{b}+U_{a}+U_{d}+U_{exc}+U_{bp}+U_{bs}+U_{cs}+U_{el},\text{\textbackslash{} \textbackslash{} \textbackslash{} \textbackslash{} \textbackslash{} \textbackslash{} \textbackslash{} \textbackslash{} \textbackslash{} \textbackslash{} \textbackslash{} }$$ (1)

where $U_{b}$, $U_{a}$, and $U_{d}$ represent the bonded interactions corresponding to bond lengths, bond angles, and dihedral angles, respectively (see Eqs. S2–S4 in S1 Text). These terms were parameterized by statistically analyzing the distributions of corresponding geometric features from experimentally resolved DNA structures (S1 Table). Notably, most resolved DNA structures are dominated by well-formed helical regions, whose geometric characteristics are not ideal for capturing the folding behavior of flexible, unstructured DNA chains. To overcome this limitation, we separately analyzed base-paired helical regions and unpaired loop regions, deriving two distinct sets of bonded parameters: Para_helix_ and Para_loop_ (S2 Table), respectively. During the folding simulations, only Para_loop_ is used to better model the conformational dynamics of single-stranded DNA. In contrast, Para_helix_ is employed exclusively in the final refinement stage to base-paired regions, ensuring the formation of canonical helical geometries with continuous base pairing.

The remaining terms in Eq. 1 describe non-bonded interactions; detailed formulations and parameterizations are provided in S1 Text. $U_{exc}$ accounts for excluded volume repulsion between CG beads (Eq. S10). $U_{bp}$, $U_{bs}$, and $U_{cs}$ describe base-pairing (Eq. S9), base-stacking (Eq. S6), and coaxial-stacking (Eq. S11) interactions, respectively. The final term, $U_{el}$ accounts for electrostatic interactions between phosphate groups (i.e., P beads), and is modeled using a Debye-Hückel approximation that incorporates both the counterion condensation theory and the tightly bound ion (TBI) model [13,58,59]:


$$U_{el}=\sum_{i<j}^{N}\frac{Q_{i}Q_{j}e^{2}}{4\pi \varepsilon_{0}\varepsilon \left(T\right)r_{ij}}e^{-\frac{r_{ij}}{l_{D}}}.\;$$ (2)

Here, e denotes the elementary charge, $r_{ij}$ is the distance between i- and j-th P beads, and *N* is the total number of P beads in a DNA chain. $l_{D}$ is the Debye length, which characterizes the ionic screening effect and depends on the ionic strength of solution. $\varepsilon_{0}$ and ε(T) denote the vacuum permittivity and the effective temperature-dependent dielectric constant, respectively [41,60]. In the present model, each P group is initially assigned a unit negative charge (−1 e). When cations are present in the environment, the effective charge of the phosphate groups is reduced. The reduced charge fraction $Q_{i}$ is defined as $Q_{i}=1-f_{i}$, where $f_{i}$ denotes the ion neutralization fraction for the *i*-th P bead. Given that complex DNA structures, particularly junctions or loop regions, exhibit distinct charge distributions compared to helical regions, the present model applies a structure-based correction to $f_{i}$ to more accurately reflect local electrostatic effects. For a solution containing pure *ν*-valent ions, $f_{i}$ is calculated as:


$$f_{i}=\frac{N\;{\bar{f}}_{i}}{\sum {\mathrm{\backslash nolimits}}_{j}^{N}e^{-\beta v\phi_{i}}}e^{-\beta v\phi_{i}},$$ (3)

where ${\overset{―}{f}}_{i}=1-\frac{b}{\left(vl_{B}\right)}$ is the average neutralization fraction derived from counterion condensation theory, *b* is the average charge spacing along the DNA backbone, and *l*_*B*_ is the Bjerrum length. The electrostatic potential *ϕ*_*i*_ at the *i*-th P bead is approximated as: $\phi_{i}=\sum_{i\neq j}^{N}\left(\frac{l_{B}Q_{j}}{r_{ij}}\right)e^{-\frac{r_{ij}}{l_{D}}}$. The reduced charge fractions $Q_{i}$ determined through an iterative procedure: (1) set $f_{i}={\overset{―}{f}}_{i}$, and compute initial $Q_{i}$; (2) calculate $\phi_{i}$ using the current $Q_{i}$; (3) update $f_{i}$ and $Q_{i}$; (4) repeat steps (2) and (3) until convergence. To reduce computational cost, $f_{i}$ is updated every 100 MC steps unless the secondary structure changes or replica exchange occurs, in which case it is updated immediately to reflect the new conformational environment. For mixed monovalent and divalent ions, $f_{i}=xf_{i}^{1}+(1-x)f_{i}^{2}$, where $f_{i}^{v}$ is the binding fraction of *v*-valent ions, and *x* denotes the contribution fractions of monovalent ions, which is derived from the TBI model x=[1+]/([1+]+α[2+]). α=(8.1−64.8/N)(5.2−ln[1+]) and [ν+] represents the bulk concentration of υ-valent ion [48]; see more details in S1 Text.

### Replica-exchange Monte Carlo simulations

The previous version of our CG model, which used MC simulated annealing algorithm for conformational sampling, successfully predicted the 3D structures of simple DNA topologies such as hairpins, duplexes, and minimal H-type pseudoknots [13]. However, it often became trapped in intermediate states when applied to more complex structures, particularly those with multi-way junctions (≥ 3-way), due to the rugged energy landscape and limited sampling efficiency [57]. To address this limitation, the present model employs a more efficient replica exchange Monte Carlo (REMC) algorithm [57,61], which enhances conformational sampling by enabling exchanges between parallel simulations (replicas) at different temperatures. Specifically, 10 replicas are simulated in parallel at temperatures ranging from 25°C to 110°C (e.g., 25°C, 31°C, 37°C, 45°C, 54°C, 64°C, 74°C, 86°C, 98°C, and 110°C). Each replica undergoes pivot-based conformational updates according to the Metropolis criterion, and adjacent replicas periodically attempt exchanges with probability p=min(1,exp(−Δ))*,* where $\Delta =\beta_{j}E\left(x_{i},T_{j}\right)+\beta_{i}E\left(x_{j},T_{i}\right)-\beta_{i}E\left(x_{i},T_{i}\right)-\beta_{i}E(x_{j},T_{j}),$ with $\beta_{i}=1/(k_{B}T_{i})$ and E(x,T)denoting the temperature-dependent potential energy of conformation *x* at temperature T. This generalized Metropolis acceptance criterion ensures that the detailed balance condition is satisfied even when the potential energy function explicitly depends on temperature [62,63].

### Identifying top-scored coarse-grained 3D structures

To identify the top-scoring 3D structures from the predicted CG structure ensemble, we first selected 1000 conformations with the lowest CG energies from the predicted ensemble at the lowest temperature (i.e., 25°C). We then applied clustering algorithms to group similar structures by calculating the RMSD values between all pairs of structures within the selected ensemble. The cluster with the largest number of structures, within a predefined RMSD threshold was identified, and its members were removed from the initial set. This process was iteratively repeated until all structures were assigned to a cluster [64], Here, a clustering threshold of 0.1 Å times the sequence length (e.g., 5 Å for a 50-residue sequence) was used here [64]. Finally, the medoids of the three largest clusters, along with the decoy with the lowest energy, were selected as the initial predictions.

### Rebuilding all-atom structure

For the top-scoring CG structures (top-1 or top-*n*), a simple structure refinement process was performed using a MC sampling at room temperature. In this process, the parameters of Para_helix_ and Para_loop_ were used to calculate the bonded interactions for base-pairing and single-stranded regions, respectively, and other nonbonded interactions were calculated as described earlier. Afterwards, the refined structure was subjected to all-atom reconstruction for practical application.

First, we constructed a small library of five non-redundant all-atom structures for each type of base pairing (G-C, A-T, C-G, T-A) as well as single nucleotides (A, G, C, T) using clustering according to their mutual RMSD values. Then, for each target CG structure, the corresponding nucleotide or base pair was aligned with same sequence fragments from the pre-built library based on CG atom positions. The fragment with the smallest RMSD to the CG atoms was selected as the all-atom replacement for that nucleotide or base pair. This process was repeated for all CG nucleotides until the complete all-atom 3D structure is constructed [14]; see S2 Fig. Finally, to eliminate potential steric clashes and chain breaks in the rebuilt all-atom structures, another refinement step was performed using the QRNAS method [65].

### Thermal stability analysis using WHAM

In addition to predicting DNA 3D structures, we applied the WHAM [66] to analyze REMC trajectories and assess DNA thermal stability by calculating the population fractions of structural states across temperatures. This approach enabled us to characterize key thermal properties such as melting temperatures and unfolding pathways. For simplicity, for the simulated DNA, we first categorized the conformations in the DNA ensemble from REMC into different structural states $S_{j}$ based on the predicted secondary structures, specifically focusing on the retention of the stems. For instance, a DNA structure with a 3-way junction can be divided into 8 states: folded (*F*), unfolded (*U*), and 6 intermediate (*I*) states corresponding to different stem configurations; see S3 Fig for details. Then, the conformational space was then discretized into bins along two reaction coordinates: structural states $S_{j}(j=1,2,\ldots .,F)$ and energy levels $E_{k}(k=1,2,\ldots ,100)$. Each bin (j,k) defines a micro-state for WHAM analysis.

The probability $p_{\left(j,k\right)}^{\circ }$ of each microstate at the target temperature $T_{i}$ was computed iteratively using [66]:


$$p_{\left(j\text{,}k\right)}^{\circ }=\frac{\sum_{i=\text{1}}^{M}n_{i\text{,}(j\text{,}k)}}{\sum_{i=\text{1}}^{M}N_{i}Z_{i}c_{i\text{,}(j\text{,}k)}};$$ (4)

$$\begin{array}{c} Z_{i}^{-\text{1}}=\sum {\mathrm{\backslash nolimits}}_{j\text{,}k}c_{i\text{,}(j\text{,}k)}p_{\left(j\text{,}k\right)}^{\circ }\text{,} \end{array}$$ (5)

where *M* is the number of replicas, $n_{i,(j,k)}$ is the count of microstate (j,k) at $T_{i}$, $N_{i}$ is the total number of conformations at $T_{i}$, and $c_{i,(j,k)}$ is the temperature bias factor. The relative fraction of each structural state $S_{j}$ at any temperature *T*, denoted $f_{S_{j}}(T)$, was obtained by summing over energy bins:


$$f_{S_{j}}\left(T\right)=\sum_{k=1}^{100}p_{\left(j,k\right)}^{\circ }.$$ (6)

For simplicity, the fractions of the folded state ($f_{F}(T)$) and unfolded state ($f_{U}(T)$) were fitted to a two-state transition model [13,67]:


$$f_{F}(T)=\frac{\text{1}}{\text{1}+e^{(T-T_{m\text{1}})/dT_{\text{1}}}};$$ (7)

$$f_{U}(T)=\text{1}-\frac{\text{1}}{\text{1}+e^{(T-T_{m\text{2}})/dT_{\text{2}}}}.$$ (8)

Here, $T_{m1}$ and $T_{m2}$ denote the melting temperatures for the folded-to-intermediate (F → I) and intermediate-to-unfolded (I → U) transitions, respectively. Detailed definitions of the structural states and further information on the WHAM procedure are provided in S1 Text. It should be point out that the temperature dependence of the potential function in the present CG model may impact the WHAM results [66]. However, the simplifications in the structural states and the two-state transition fitting likely mitigate its effects.

## Results and discussion

In this work, we applied our newly developed CG model to predict the 3D structures of complex DNA molecules, including those with three-way and four-way junctions, extending beyond the simpler ssDNA and dsDNA structures. We then evaluated the thermal stability of these complex DNA structures in both monovalent and divalent ion solutions, surpassing the simple ssDNA, dsDNA, and pseudoknot structures explored in our previous work [13]. Finally, we performed a comprehensive analysis of the thermally unfolding pathways for DNA molecules containing three-way and four-way junctions.

### Predicting 3D structures of DNA with multi-way junctions

#### Overview of 3D structure prediction framework.

As shown in Fig 1, the present model predicts DNA 3D structures from its sequence through five key steps: (1) An initial random conformation of the DNA chain is generated based on the given sequence, utilizing bonded potential $U_{bond}$ and the excluded-volume potential $U_{exc}$ (Eq. 1); (2) This conformation undergoes a REMC simulation for conformational sampling, during which only the parameters of Para_loop_ for bonded potential are applied to simulate the flexible nature of the DNA chain; (3) Top structures from the REMC ensemble at the lowest temperature (e.g., 25°C) are selected using the CG energy and a clustering algorithm [68]; (4) A further structure refinement (i.e., MC simulation at 25°C) is performed on the top-scoring conformations by applying two distinct sets of bonded potential parameters: Para_loop_ for the loop regions and Para_helix_ for the base-pairing regions, enabling a more accurate representation of the helical geometry of the stems [69]; (5) Finally, the refined CG structures are reconstructed into all-atom structures.

To evaluate the performance of the present model, two primary metrics (RMSD and F1-score) are calculated for the predicted structures. The RMSD metric, including both the RMSD of the top-ranked structure (top-1 RMSD) and the minimum RMSD observed in the ensemble (RMSDmin), quantifies the global deviation between the predicted and native structures [70,71]. Both top-1 RMSD and RMSDmin are used to assess the structural prediction accuracy of the model. In addition to the RMSD of the top-1 structure (i.e., top-1 RMSD), the structure with minimum RMSD (i.e., RMSD_min_) from the predicted structure ensemble is also used to assess the performance of model. To assess the accuracy of predicted DNA secondary structures, the F1-score, which measures the consistency of base-pairing interactions between the predicted and native structures [72,73], is also calculated by F1=2×PR×SN/(PR+SN), where precision (PR) is defined as PR=TP/(TP+FP) and sensitivity (SN) as SN=TP/(TP+FN), with TP, FP, and FN denoting true positives, false positives, and false negatives, respectively.

### Validation using experimental DNA junction structures

**Fig 2:**
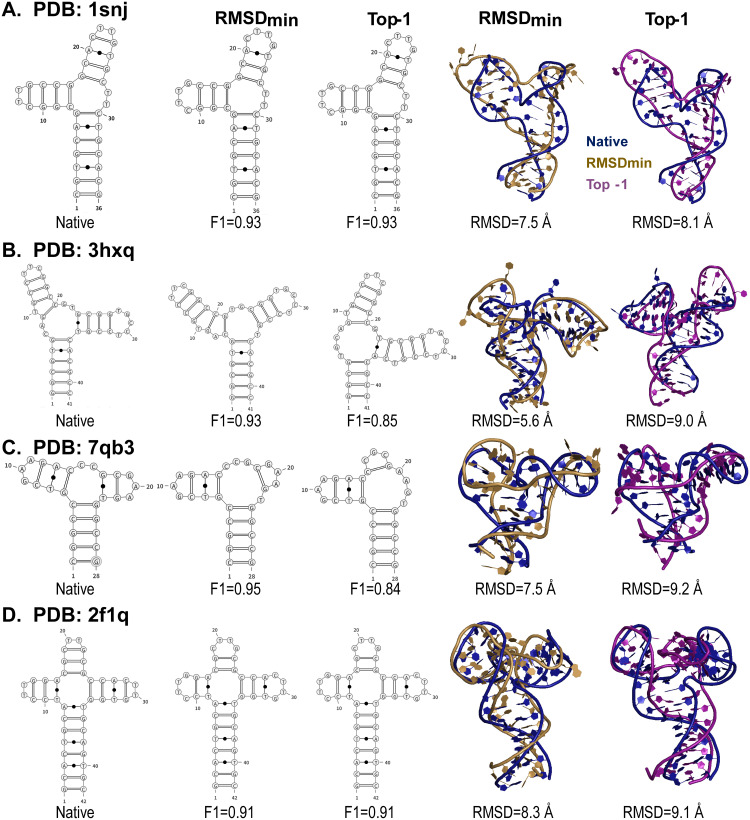
Comparison of predicted secondary and 3D structures of DNA junctions with RMSD_min_ and top-1 RMSD using our CG model. **(A-D)** F1 scores and RMSD values for the minimum RMSD and top-1 RMSD predicted structures of DNAs with three-way junctions (A-C) and four-way junctions (D). The predicted 3D structures with minimum RMSD (brown) and top-1 RMSD (pink) are superimposed on the corresponding native structures (blue). Secondary structures were visualized using VARNA [92], and 3D structures were rendered in PyMOL [93].

In this work, we predicted 3D structures of four DNAs containing three-way (PDB IDs: 1snj, 3hxq, 7qb3) and four-way (PDB ID: 2f1q) junctions under standard salt conditions (1M NaCl) [74–76]. As shown in Fig 2, both secondary and tertiary structures were compared with experimentally determined structures. The predicted secondary structures (parsed by DSSR from 3D models with Top-1 RMSD and RMSD_min_) largely recapitulate the native base-pairing patterns across all four DNAs, as evidenced by high F1-scores ranging from 0.84 to 0.95. This highlights the ability of model to recover key structural motifs from sequence alone. However, discrepancies remain in certain local and non-canonical base pairs (e.g., C11-G14/C21-G24 in 1snj, G23-T35 in 3hxq, G17-T23 in 7qb3, and C11-G14/C19-G22/C28-G31 in 2f1q). These deviations mainly arise from model assumptions that restrict base pairing to nucleotides separated by at least three positions and exclude non-canonical interactions such as G-T pairs, which should be further improved in future.

All four DNA molecules lack long-range tertiary base pairs (Fig 2), which poses a significant challenge for accurately predicting the relative orientations between stems connected by junctions. Nevertheless, the best predicted 3D structures (RMSD_min_) achieve RMSD values ranging from 5.6 to 8.3 Å, while the top-ranked models (Top-1) fall within 8.1-9.2 Å. These results likely benefit from the coaxial stacking interactions in the present model, as well as efficient sampling enabled by the pivot move-based algorithm [56,57].

Collectively, these results demonstrate that the present model can accurately capture both secondary and tertiary structures of DNAs with multi-branched architectures, offering a powerful tool for modeling DNA structures beyond simple duplexes/hairpins.

### Comparison with existing methods

To benchmark the present model, we compared it with two representative methods for DNA 3D structure prediction: 3dRNA/DNA [77] and AlphaFold3 [18], using only sequence information as input for all approaches. For 3dRNA/DNA, we used its webserver (http://biophy.hust.edu.cn/new/3dRNA/create) with the default ‘RNAfold’ settings for secondary structure prediction and the ‘Optimization’ option for 3D structure refinement. For AlphaFold3, end-to-end structure predictions were obtained via its webserver (https://alphafoldserver.com/).

**Fig 3:**
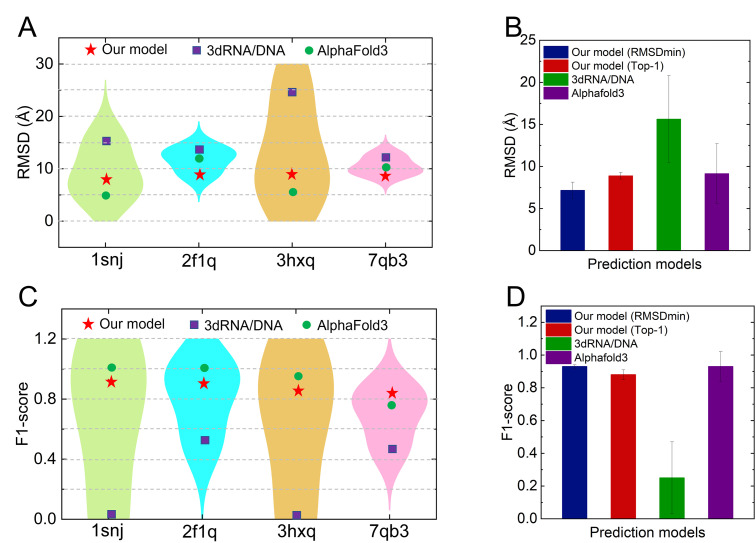
Performance comparison of our CG model with 3dRNA/DNA and AlphaFold3 in predicting the 3D structures of DNA molecules with multi-way junctions. **(A, C)** RMSD values (A) and F1 scores (C) for four DNAs with multi-way junctions predicted by our CG model, 3dRNA/DNA, and AlphaFold3. **(B, D)** Average RMSD values (B) and F1 scores (D) across the four DNAs for each method. Error bars represent the standard deviation of the RMSD and F1 score values.

Fig 3A and 3C display the RMSDs and F1-scores of the top-ranked (Top-1) structures predicted by three methods for each DNA molecule. For the four DNAs, the RMSDs predicted by the present model are consistently lower than those obtained from 3dRNA/DNA, with two of the predictions outperforming those from AlphaFold3 (Fig 3A). As shown in Fig 3B, for the four DNAs, the average RMSD for the top-ranked (Top-1) predictions from our model is approximately 8.9 Å, representing an improvement of about 43.6% compared to 3dRNA/DNA (~15.6 Å). This improvement is largely attributed to the significantly higher accuracy of our predicted secondary structures (Fig 3C and 3D), highlighting the limitations of fragment assembly approaches that heavily depend on secondary structure inputs [26]. AlphaFold3 achieves an average RMSD of ~9.2 Å, which is comparable to our Top-1 results, but still higher than the best structures predicted by our model (RMSD_min_). It is important to note that the experimental structures of these four DNAs were released between 2004 and 2021, while AlphaFold3 was trained on all macromolecular structures available in the PDB prior to January 2023 [18]. This suggests that these DNAs were likely included in its training set, which may explain the near-perfect agreement between its predicted and native base-pairing patterns (Fig 3C and S4 Table). Nevertheless, the performance of AlphaFold3 on these DNAs is notably lower than its typical accuracy (< 3 Å) for protein structure prediction [18,19]. This discrepancy underscores the limited availability of high-quality DNA structural data and highlights the continued need for physics-based modeling approaches.

### Predicting thermal stabilities of DNA junctions under ion conditions

DNA functionality depends not only on its 3D structure but also on its thermal stability [13]. Beyond 3D structure prediction, the present model enables quantitative prediction of the thermal unfolding behavior of DNAs containing complex motifs such as three-way (3WJ) and four-way junctions (4WJ) in monovalent and divalent ionic environments.

#### Predicting thermal stabilities of DNA with multi-way junctions.

We tested the present model on six DNAs, three with 3-way junctions and three with 4-way junctions, whose sequences and structural properties are summarized in Table 1 and S4 Fig. As described in the *Materials and Methods* section, for each DNA, we combined REMC simulations with the WHAM to compute two distinct melting temperatures: one corresponding to the transition from the folded state to an intermediate state ($T_{m1}$), and the other from an intermediate state to the unfolded state ($T_{m2}$).

**Table 1:** The predicted melting temperature ($T_{m1}$^*a*^ and $T_{m2}$^*b*^) of 6 DNAs by our present model.

DNAs	Type	Refs	Length (nt)	Predicted $T_{m1}$/$T_{m2}$ (°C)	Expt. $T_{m1}$/$T_{m2}$ (°C)
3WJ	Three-way	[78]	37	34.4^*c*^/64.6^*c*^	33.6^*c*^/70.3^*c*^
L-3WJ	Three-way	[78]	37	36.2^*c*^/82.5^*c*^	35.8^*c*^/87.6^*c*^
R-3WJ	Three-way	[78]	37	50.4^*c*^ (54.2^*d*^)/73.5^*c*^	54.6^*d*^/77.9^*c*^
4WJ	Four-way	[78]	53	28.6^*c*^/72.1^*c*^	51.2^*e*^/65.0^*e*^
L-4WJ	Four-way	[78]	52	32.6^*c*^/83.1^*c*^	52.4^*f*^/87.9^*f*^
R-4WJ	Four-way	[78]	52	31.7^*c*^/81.3^*c*^	52.8^*g*^/72.9^*g*^

^a^$T_{m1}$ and ^*b*^$T_{m2}$ are the melting temperatures for the transitions from folded state to intermediate state and from intermediate state to unfolded state, respectively. ^*c*^The melting temperature $T_{m1}$ (3 stems transformed into 2 stems) and $T_{m2}$ (1 stem transformed into 0 stem). ^*d*^The melting temperature $T_{m1}$ (3 stems transformed into 1 stem). ^*e*^The melting temperature $T_{m1}$ (4 stems transformed into 1.5 stem) and $T_{m2}$ (1.5 stem transformed into 0 stem). ^*f*^ The melting temperature $T_{m1}$ (4 stems transformed into 1.5 stem) and $T_{m2}$ (1 stem transformed into 0 stem). ^*g*^ The melting temperature $T_{m1}$ (4 stems transformed into ~1.5 stem) and $T_{m2}$ (~1.5 stem transformed into 0 stem).

**Fig 4:**
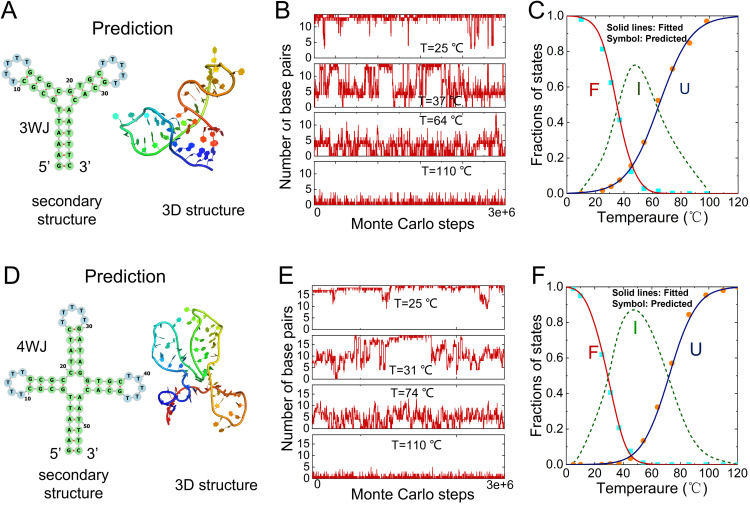
Predicted melting behavior of DNAs with 3-way (3WJ) and 4-way (4WJ) junctions using our CG model. **(A, D)** Predicted secondary and 3D structures of the 3WJ (A) and 4WJ (D) by our CG model. **(B, E)** Time evolution of the base-pair fractions for 3WJ (B) and 4WJ (E) at different temperatures (110°C, 74°C, 45°C, 25°C, from bottom to top). **(C, F)** Temperature-dependent fractions of the folded (F, green) and unfolded (U, red) states for 3WJ (C) and 4WJ (F) in 1M NaCl.

As illustrated in Fig 4A–4C, taking a 37-nt DNA (the 3WJ with sequence of 5’-GAAATTGCGCTTTTTGCGCGTGCTTTTTGCACAATTTC-3’) as an example [78], the REMC simulations at 0.1M NaCl yield predictions of its secondary structure (Fig 4A). Based on the base-pairing configurations observed in REMC trajectories at different temperatures (Fig 4B), the conformational ensemble can be grouped into eight microstates, including the folded state, the unfolded state, and six intermediate states (S3 Fig). WHAM was then used to compute the fractional populations of each state across temperatures. Finally, using the temperature-dependent fractions of the folded and unfolded states ($f_{F}$ and $f_{U}$), we fit two-state transition models to extract the two melting temperatures (Fig 4C), representing the folded-to-intermediate and intermediate-to-unfolded transitions, respectively. The predicted two melting temperature: $T_{m1}$ = 34.4°C and $T_{m2}$ = 64.6°C, are close to experimental data ($T_{m1}$ = 33.6°C and $T_{m2}$ = 70.3°C), with deviations within ~2–6 °C (Table 1). In addition, the Fig 4D–4F also present the predicted results for another DNA structure featuring a 4-way junction (4WJ, 53nt; sequence: 5’-GAAATTGCGCTTTTTGCGCATATCTTTTTGATAGGTGCTTTTTGCACAATTTC-3’) [78]. Compared to the 3-way junction case, this system exhibits more complex structural fluctuations (Fig 4E), but follow similar thermal behavior. The predicted melting temperatures are $T_{m1}$ = 28.6°C and $T_{m2}$ = 72.1°C, showing notable deviation from the experimental values of $T_{m1}$ = 51.2°C and $T_{m2}$ = 65.0°C, especially in the first transition temperature. This discrepancy is likely due to the current simplification of model, which considers only folded and unfolded states while neglecting the stability differences among intermediate states.

Table 1 further summarizes the predictions for four additional DNAs (L-3WJ, R-3WJ, L-4WJ, and R-4WJ) [78]. For the three DNAs with three-way junctions (3WJ, L-3WJ, and R-3WJ), the predicted melting temperatures ($T_{m1}$ and $T_{m2}$) show good agreement with experimental data. The mean deviations are ~1.8°C for $T_{m1}$ and ~4.9°C for $T_{m2}$, suggesting that the model reliably captures the thermal stability of DNA with three-way junctions [78]. Additionally, for R-3WJ, the predicted melting temperature $T_{m1}$ of 50.4°C is lower than experimental value (54.6°C). This could be because the experimental $T_{m1}$ corresponds to the melting of two stems, while the model defined $T_{m1}$ refers to the melting of a single stem. To address this discrepancy, we redefined the intermediate state containing two stems as the folded state, yielding a revised model prediction of 54.2 °C, closely matching the experimental result.

For DNAs with four-way junctions (4WJ, L-4WJ, and R-4WJ), a similar challenge arises. In experiments, the melting transitions often involve the cooperative melting of multiple stems [78], whereas our model treats transitions at the single-stem level. As a result, the predicted $T_{m1}$ values are consistently lower than the experimental ones, while $T_{m2}$ values tend to be higher (Table 1). This is because the experimentally determined melting temperatures likely correspond to more stable intermediate states involving more than one stem, whereas our model simplifies these to a single-stem melting event. Nevertheless, the overall thermal transition behavior is well captured, supporting the predictive reliability of the model.

#### Effect of monovalent ion on the stability of DNAs with multi-way.

DNA 3D structures and their thermal stabilities are highly sensitive to the ionic conditions due to the polyanionic nature of the DNA backbone [44,45,79]. In this work, we examined the effects of the monovalent salt (Na^+^) on DNA stability using two representative systems: a 3-way junctions (3WJ) and a 4-way junctions (4WJ) [78].

**Fig 5:**
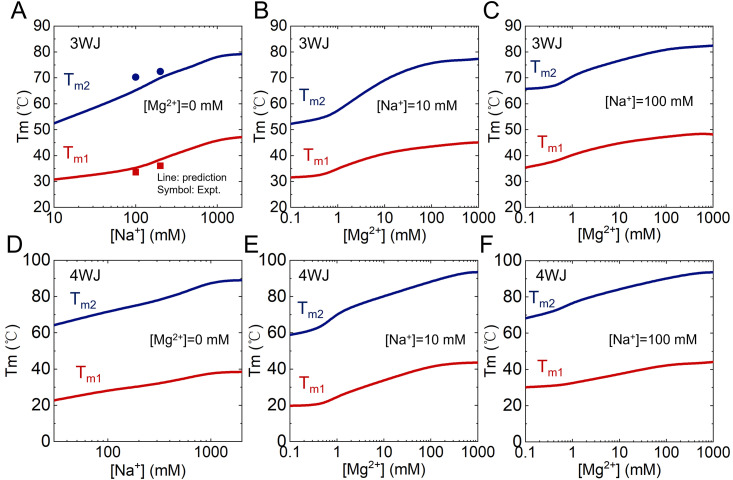
Comparison of predicted (lines) and experimental (symbols) melting temperatures for 3WJ and 4WJ. **(A)** Melting temperatures of the folded-to-intermediate (F → I, $T_{m1}$) and intermediate-to-unfolded (I → U, $T_{m2}$) transitions for 3WJ as a function of [Na^+^]. **(B, C)** Melting temperature ($T_{m1}$ and $T_{m2}$) for 3WJ as a function of [Mg^2+^] at fixed [Na^+^] of 10 mM (B) and 100 mM (C). **(D)** Melting temperature for 4WJ of the F → I ($T_{m1}$) and I → U ($T_{m2}$) transitions as a function of [Na^+^]. **(E, F)** Melting temperature of 4WJ as a function of [Mg^2+^], with [Na^+^] fixed at 10 mM (E) and 100 mM (F).

We further extended our predictions to a wider range of Na^+^ concentrations (see S5 and S6 Tables). As shown in Fig 5A, the stabilities of both the folded and intermediate hairpin structures increase with rising [Na^+^], which can be attributed to the stronger electrostatic screening at higher [Na^+^], particularly beneficial for compactly folded or intermediate conformations. Interestingly, we observed that the increase in $T_{m2}$ (unfolding of intermediate hairpin to single strand) with Na^+^ concentration is slightly pronounced than that of $T_{m1}$ (transition from fully folded to intermediate state). This is likely due to the fact that the difference in charge density between the intermediate hairpin and fully unfolded state is typically larger than that between the fully folded state and the intermediate state with a single stem. As shown in S5A Fig, for the 3WJ, the average radius of gyration (Rg) of the folded state is 16.9Å, which is only slightly smaller than that (17.6Å) of the intermediate state with one melted stem. In contrast, the Rg of the fully unfolded conformations (25.2Å) is significantly larger than that of the intermediate hairpin state (22.9Å), indicating a more extended and less compact structure. In addition, as shown in S5B Fig, the average ion neutralization fraction (f) of the folded state (0.54) is only marginally higher than that of the one-stem-melted intermediate (0.48), whereas the fully unfolded conformations have a notably lower *f* value (0.37) than the hairpin intermediate (0.46). Similarly, for the 4WJ (S5C and S5D Fig), the folded state exhibits an average Rg of 19.2 Å, also slightly smaller than that of the one-melted-stem intermediate state (19.8 Å). In contrast, the fully unfolded conformations (31.4Å) exhibit a much larger Rg compared to the intermediate hairpin state (27.6Å). Regarding ion neutralization, the folded state shows a slightly higher f (0.62) than the single-melted-stem intermediate (f = 0.60), whereas the fully unfolded state displays a significantly lower value f (0.47) relative to the three-stem-melted intermediate (0.52). These suggests that the electrostatic stabilization effect of Na^+^ is more prominent during the transition from intermediate to fully unfolded states, thereby explaining the sharper increase in $T_{m2}$ with ion concentration.

#### Effect of divalent ion on the stability of DNAs with multi-way junction.

To further investigate the ionic effects on DNA folding, we analyzed the predicted melting temperatures ($T_{m1}$ and $T_{m2}$) of DNA with multi-way junctions across a wide range of [Mg^2+^], while fixing [Na^+^] at 10 mM (Fig 5B) and 100 mM (Fig 5C), respectively. The results reveal that the present model successfully captures the competitive and cooperative interactions between monovalent (Na^+^) and divalent (Mg^2+^) ions in modulating the stability of DNA with three-way junctions (3WJ). At low [Mg^2+^] (e.g., 1 mM), the stability of 3WJ is dominated by the background Na^+^ concentration, and the predicted temperatures ($T_{m1}$ and $T_{m2}$) closely resemble those observed in pure Na^+^ solutions. As [Mg^2+^] increases (~>1 mM), the stability of the 3WJ structure is significantly enhanced due to the stronger charge neutralization of Mg^2+^. This stabilizing effect saturates at higher [Mg^2+^] (~>100 mM). This phenomenon is due to the anti-cooperative binding between Na^+^ and Mg^2+^, coupled with the more efficient stabilization provided by Mg^2+^. A similar trend is observed for four-way junctions (4WJ), as shown in Fig 5D–5F, further supporting the robustness of the model in describing the thermodynamic effects of divalent cations.

These findings indicate that the present model can quantitatively capture the influence of both monovalent and divalent ions on the thermal stability of complex DNA junction structures. However, recent studies [47,80,81] have shown that at very high ionic concentrations, excessive ion binding can lead to overcharging effects, which in turn destabilize DNA structures. Since the implicit ion model used in our model assumes a maximal screening effect, effectively neutralizing the DNA backbone charges, it is inherently unable to capture such overcharging phenomena. This limitation suggests that incorporating explicit ion representations, particularly for multivalent ions, may be essential for accurately modeling DNA stability under extreme ionic conditions [41,54,55].

### Thermally unfolding pathway of DNAs with multi-way junctions

Understanding the unfolding pathways of DNA multi-way junctions is essential for elucidating their structure-function relationships [78]. The present model not only accurately predicts the melting temperatures of DNA three- and four-way junctions but also reveals the detailed thermally induced transitions among folded, intermediate, and unfolded states based on their temperature-dependent fractions calculated from REMC trajectories using WHAM. In this work, we focus on two typical DNA with multi-way junctions structures 3WJ (DNA with three-way junction) and 4WJ (DNA with four-way junction) to analyze their unfolding pathways beyond the minimal ssDNA and dsDNA [13]. Here, the intermediate states are denoted as I1, I2,..., corresponding to the number of melting stems, while states without labels or those labeled with ‘′’, ‘′′’, ‘′′′’, etc., represent states with the highest, second-highest, third-highest fraction, and so on.

#### For a DNA with three-way junctions (3WJ).

**Fig 6:**
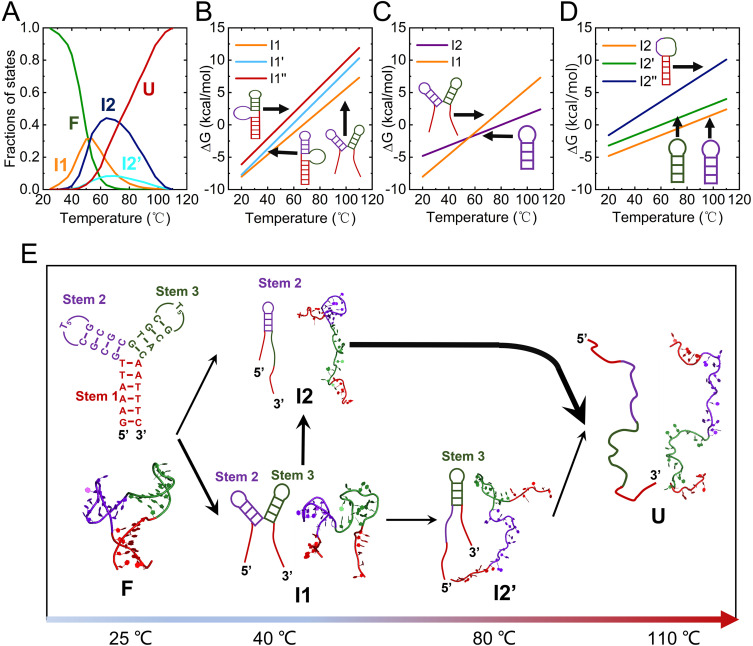
Predicted thermally unfolding pathway of 3WJ at 1 M [Na^+^] using our CG model. **(A)** Temperature-dependent population fractions of different structural states: fully folded (F), intermediate (I1, I2, I2′), and fully unfolded (U). F represents the fully folded conformation, I1 corresponds to the intermediate state with Stems 2 and 3 melted, I2/I2′ represent the hairpin intermediates with Stem 2/Stem 3, and U denotes the fully unfolded structure. **(B-D)** Temperature-dependent free energies of various intermediate states (I1, I1′, I1′′, I2, I2′, and I2′′′) calculated using Mfold. **(E)** Schematic representation of the structure transitions along the unfolding pathway inferred from the state fractions shown in panel (A).

At 1 M NaCl, the 3WJ structure exhibits a stepwise unfolding mechanism involving three key intermediates (I1, I2, and I2′) (Fig 6). As shown in Fig 6A, at low temperatures (<~30°C), the 3WJ remains predominantly in the fully folded state (F). As the temperature increases from ~30°C to ~50°C, the fraction of F decreases from ~99% to ~33%, while the fractions of intermediate states I1, I2, and I2′ gradually increase to ~31%, ~ 30%, and ~5%, respectively. Between ~50°C and ~70°C, the fractions of the F and I1 further decline to ~2% and ~9%, whereas I2 and I2′ reach their peak values of ~45% and ~10%, respectively. At ~110°C, the system transitions into the unfolded state (U). These observations suggest that the dominant unfolding pathway of the 3WJ follows F → I1 → I2 → U, with I2 being the most populated intermediate at ~70°C. Additionally, two minor pathways are identified: F → I2 → U and F → I1 → I2′→U, with the former having a slightly higher flux than the latter.

Given that the inferred unfolding pathways of the 3WJ have not been experimentally validated, we employed the well-established nearest-neighbor thermodynamic model (i.e., Mfold [67,82]) to independently estimate the free energies of eight structural states (see S3 Fig) to further support our proposed mechanism. At ~50°C, the relative free energy differences between the I1 and I1′ states $\left(\Delta \Delta G_{I1,I1\prime }=\Delta G_{I1}-\Delta G_{I1\prime }\right)$ was ~ -1.2 kcal/mol, and $\Delta \Delta G_{I1,I1\prime \prime }$ = ~ -2.8 kcal/mol, indicating that I1′ and I1′′are less stable than I1. This agrees with the higher observed population of I1 compared to I1′ and I1′′ at this temperature. Next, we examined the I2 state (stems 1 and 3 melted). Its free energy is higher than that of I1 below ~50°C but becomes lower at higher temperatures, with $\Delta \Delta G_{I2,I1}$ = ~ 0.5 kcal/mol at 50°C and ~-1.3 kcal/mol at 70°C. This suggests that I2 becomes more populated as temperature increases, supporting the major unfolding pathway F → I1 → I2 → U. We also evaluated states with two stems melted, including I2′ (stems 1 and 2 melted) and I2′′ (stems 2 and 3 melted), which have negligible populations. Notably, I2′′ exhibited significantly higher free energy than I2 and I2′, consistent with its negligible fraction.

#### For a DNA with four-way junction (4WJ).

**Fig 7:**
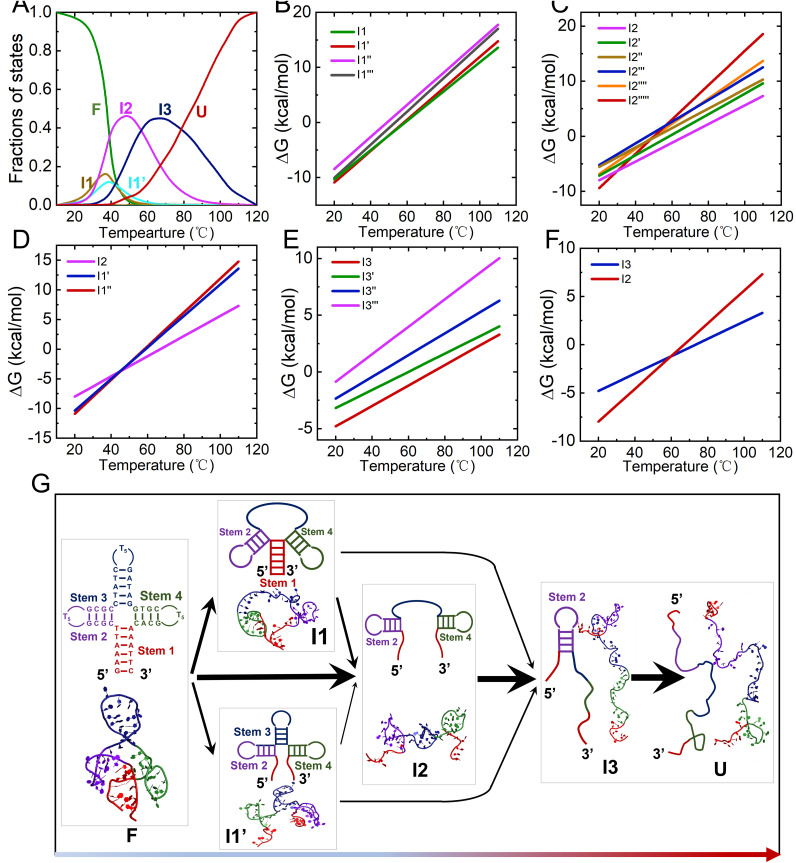
Predicted thermal unfolding pathway of 4WJ at 1 M [Na^+^] using our CG model. **(A)** Temperature-dependent fractions of the folded state (F), intermediate states (I1, I1′, I2, I3), and unfolded state (U) during the thermally unfolding. I1: with Stems 1, 2, and 4, I1′: with Stems 2, 3, and 4, I2: with Stems 2 and 4, I3: with Stem 2. **(C-F)** Free energies of various intermediate states (I1, I1′, I1′′, I1′′′, I2, I2′, I2′′, I2′′′, I2′′′′, I2′′′′′, I3, I3′, I3′′, and I3′′′) as functions of temperature calculated using Mfold. **(G)** Schematic representation of DNA structure transitions along the unfolding pathway.

The 4WJ displays a more complex landscape, with up to sixteen intermediate states (S6 Fig). Based on our model predictions, we calculated the population fractions of key structural states across temperatures (Fig 7A). At low temperatures (<~20°C), the 4WJ predominantly adopts the fully folded state (F). As temperature increases to ~40°C, the fraction of F decreases to ~30%, accompanied by a rise in intermediate states I1 (Stem 3 melted), I1′ (Stem 1 melted), and I2 (Stems 1 and 3 melted), reaching ~14%, ~ 13%, and ~38%, respectively. At ~50°C, I2 becomes the dominant intermediate (~48%), while F, I1, and I1′ nearly vanish. With further heating, I2 transitions into I3 (three stems melted), which peaks (~47%) around 70°C. Eventually, the system reaches the fully unfolded state (U) at ~120°C. Based on these results, the primary unfolding pathway is proposed as F → I2 → I3 → U, with additional minor routes: F → I1 → I2 → I3 → U, and F → I1′→I2 → I3 → U.

To validate the model-predicted pathway, we computed the free energies of key intermediate states using Mfold [82]. For accurate secondary structure prediction for Mfold, loop sequences were replaced with ‘X’. We first analyzed single-stem-melted states at 1 M NaCl and ~30°C. The relative free energy differences were: ($\Delta \Delta G_{I1,I1\prime }$ = ~ -0.4 kcal/mol, $\Delta \Delta G_{I1,I1\prime \prime }$ = - ~ 1.0 kcal/mol, $\Delta \Delta G_{I1,I1\prime \prime \prime }$ = - ~ 2.5 kcal/mol, $\Delta \Delta G_{I1\prime ,I1\prime \prime }$ = - ~ 0.6 kcal/mol, and $\Delta \Delta G_{I1\prime ,I1\prime \prime \prime }$ = ~ -2.2 kcal/mol, indicating that I1′′ and I1′′′ are much less stable than I1 and I1′, which is consistent with their low populations. We then examined the I2 state (Stems 1 and 3 melted). At ~30°C, it is less stable than I1/I1′ ($\Delta \Delta G_{I2,I1}=\sim 1.8$ kcal/mol and $\Delta \Delta G_{I2,I1\prime }=\sim 1.4$ kcal/mol), but becomes more favorable at ~50°C (both were ~ -0.6 kcal/mol), supporting its dominance in the major pathway. States with two (e.g., I2′, I2′′) or three (e.g., I3′, I3′′, I3′′′) stems melted exhibit significantly higher free energies and negligible populations (Fig 7), consistent with their minor roles. Overall, the free energy landscape confirms that unfolding proceeds primarily via F → I2 → I3 → U, with I2 serving as a key thermodynamic intermediate.

## Conclusion

In conclusion, this study presents a significant advancement in modeling the 3D structure and thermal stability of complex DNAs, with a particular focus on multi-way junctions. By refining the electrostatic energy terms of our CG model, incorporating the REMC algorithm, and using the WHAM, we have extended the applicability of model to three-way and four-way DNA junctions under both monovalent and divalent ion conditions. The main contributions of this work are as follows:

1. Accurate structure predictions: The refined CG model can predict near-native structures of complex DNAs, including three-way and four-way junctions, from sequence. The predictions align well with results from state-of-the-art models such as 3dRNA/DNA and AlphaFold3, demonstrating the capacity of model to capture the intricate geometry of DNAs with multi-way junctions.
2. Reliable thermal stability profiling: The model can reproduce the thermal stability of DNA junctions with different sequences and topologies. Notably, it successfully captures the effects of both monovalent and divalent ions on DNA junction stability, showing strong agreement with experimental data. This demonstrates the capacity of the model to simulate biologically relevant conditions.
3. Insight into thermally unfolding pathways: Our analysis reveals that the thermal unfolding of multi-way junctions proceeds via discrete intermediate states, whose relative stabilities govern the unfolding trajectories. These findings elucidate the thermodynamic landscape of DNA junctions and offer a detailed view of their transition pathways.

Despite these advances, several limitations remain. First, the model does not account for non-canonical base pairings (e.g., G-G, G-T, A-A, A-G) [83,84], which are crucial for the stability and structural features of functional DNA structures, such as DNA triplexes, and G-quadruplexes [85,86], meaning that the current version of the model is unable to predict these complex structures. Second, while the model incorporates the effects of mono-/divalent ions through the CC theory and TBI model, the implicit ion model could be insufficient to capture the interactions between multi-valent ions (e.g., Mg^2+^ and Go^3+^) and DNA, particularly the specific binding of these ions. Such interactions are critical for stabilizing complex structures like DNA G-quadruplexes [87,88], making it necessary to incorporate explicit treatment of divalent ions in future improvements to the model [54,55]. Third, the top-ranked structure predicted by the present model typically does not correspond to the most native-like structure within the predicted ensemble, highlighting the need for further development of DNA scoring function to identify the near-native structure models [89]. Finally, the cellular environment of DNA involves not only ions but also other macromolecules (e.g., proteins and RNAs) and small molecules (e.g., ligands). This crowded environment and its interactions with DNA can influence DNA structural folding. Effectively accounting for these interactions to study DNA folding in a cell-like environment remains a significant challenge [53,90,91].

Although there are limitations, our refined CG model provides a powerful and reliable framework for studying the 3D structures and stability of complex DNA configurations in the presence of physiologically relevant ions. The insights gained from analyzing the thermally unfolding pathways of DNA junctions contribute to a deeper understanding of DNA stability and the mechanisms underlying their biological functions, providing a strong foundation for future studies in the field of DNA biophysics.

## Supporting information

S1 Fig **(A) The schematic diagram for the formation of one base stacking between base pairs (*i, j*) and (*i* + 1,** ***j*-1); (B) The conformational entropy changes ΔS**_**c**_ **for the formation of base-pairs stacking at different location *i* (symbols), and the average value of ΔS**_**c**_ **(line).**
(TIF)

S2 Fig The schematic diagram of the rebuilding of coarse-grained DNA structures into all-atom ones in our present model. (TIF)

S3 FigThe eight structural states of 3WJ, including a folded state, six intermediate states, and a unfolded state.Here, F state: Stems 1, 2, and 3 retained, I1: Stem 1 resolved, I1′: Stem 3 resolved, I1′′: Stem 2 resolved, I2: Stems 1 and 3 resolved, I2′: Stems 1 and 2 resolved, I2′′: Stems 2 and 3 resolved, U: All stems resolved.(TIF)

S4 Fig The sequence and secondary structure of 6 DNAs with multi-way junctions employed in this paper. (TIF)

S5 Fig The average radius of gyration (Rg) and ion neutralization fraction of different structural states for 3WJ and 4WJ. (A,B) The average Rg (A) and ion neutralization fraction (B) of folded state (F), intermediate states I1*/I2* (two stems retained/one stem retained), unfolded state (zero stem retained) for 3WJ. (C,D) The average Rg (A) and ion neutralization fraction (B) of folded state (F), intermediate states I1*/I3* (three stems retained/one stem retained), unfolded state (zero stem retained) for 4WJ.
(TIF)

S6 FigThe sixteen structural states of 4Wj, including a folded state, fourteen intermediate states, a unfolded state.Here F: All stem retained, I1: Stem 3 resolved, I1′: Stem 1 resolved, I1′′: Stem 4 resolved, I1′′′: Stem 2 resolved, I2: Stems 1 and 3 resolved, I2′: Stems 1 and 4 resolved, I2′′: Stems 2 and 3 resolved, I2′′′: Stems 1 and 2 resolved, I2′′′′: Stems 2 and 3 resolved, I2′′′′′: Stems 2 and 4 resolved, I3: Stems 1,3, and 4 resolved, I3′: Stems 1, 2, and 3 resolved, I3′′: Stems 1, 2, and 4 resolved, I3′′′: Stems 2, 3, and 4 resolved, U: All stems resolved.(TIF)

S1 Table The PDB codes of 138 DNAs used in our statistical analysis for CG force field. (XLSX)

S2 Table The parameters of bonded potentials of CG force field. (XLSX)

S3 Table The parameters for the energy functions of base pairing and base stacking. (XLSX)

S4 Table The predicted RMSD and F1-socre by three prediction models for 4 DNAs. (XLSX)

S5 Table The predicted temperature of 3WJ at extensive ion concentration by our present model. (XLSX)

S6 Table The predicted temperature of 4WJ at extensive ion concentration by our present model. (XLSX)

S1 Text Detailed description of the coarse-grained force field of the present model and the Weighted Histogram Analysis Method. (DOCX)
