# Supplementary figures and images for: 3D structure and stability prediction of DNA with multi-way junctions in ionic solutions
Source: PLoS Comput Biol. 2025 Aug 18;21(8):e1013346. doi: 10.1371/journal.pcbi.1013346 (PMC12373291; doi:10.1371/journal.pcbi.1013346)

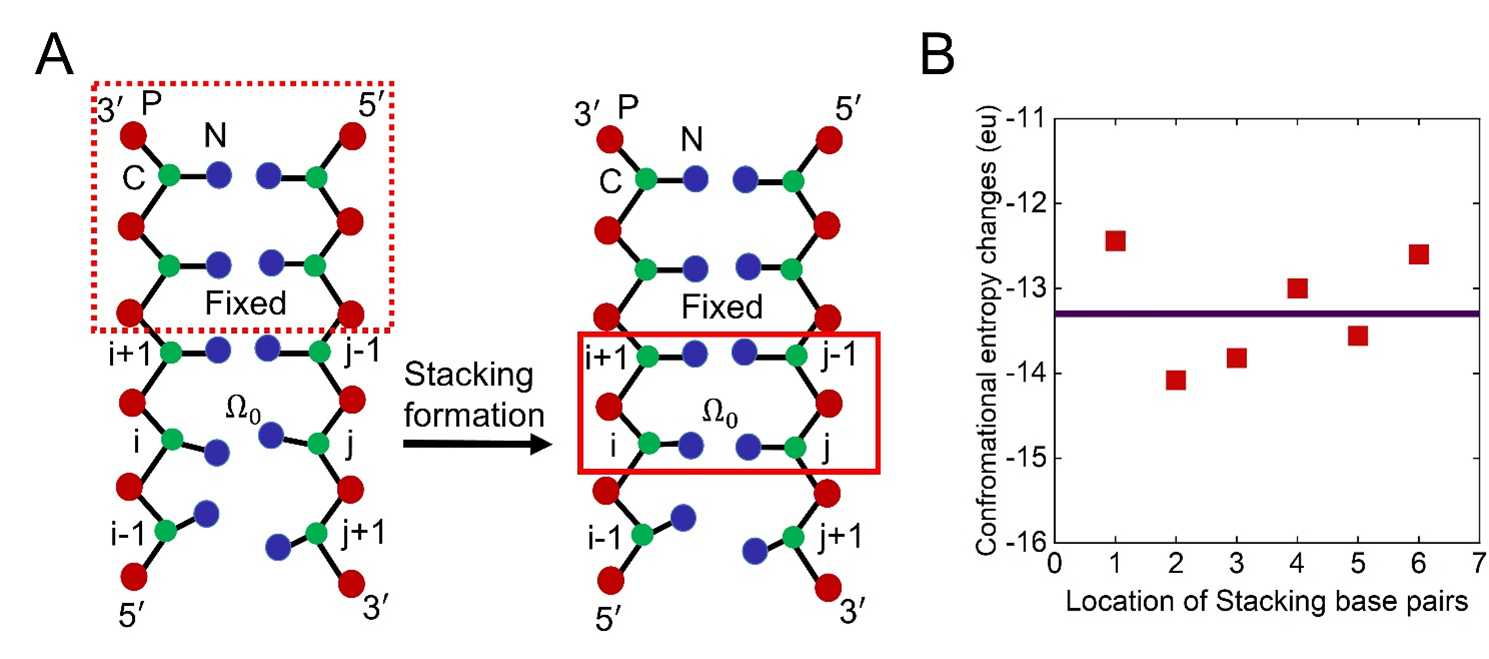

Supplement: S1 Fig — (A) The schematic diagram for the formation of one base stacking between base pairs (i, j) and (i + 1, j-1); (B) The conformational entropy changes ΔSc for the formation of base-pairs stacking at different location i (symbols), and the average value of ΔSc (line). (TIF) [file pcbi.1013346.s001.tif]

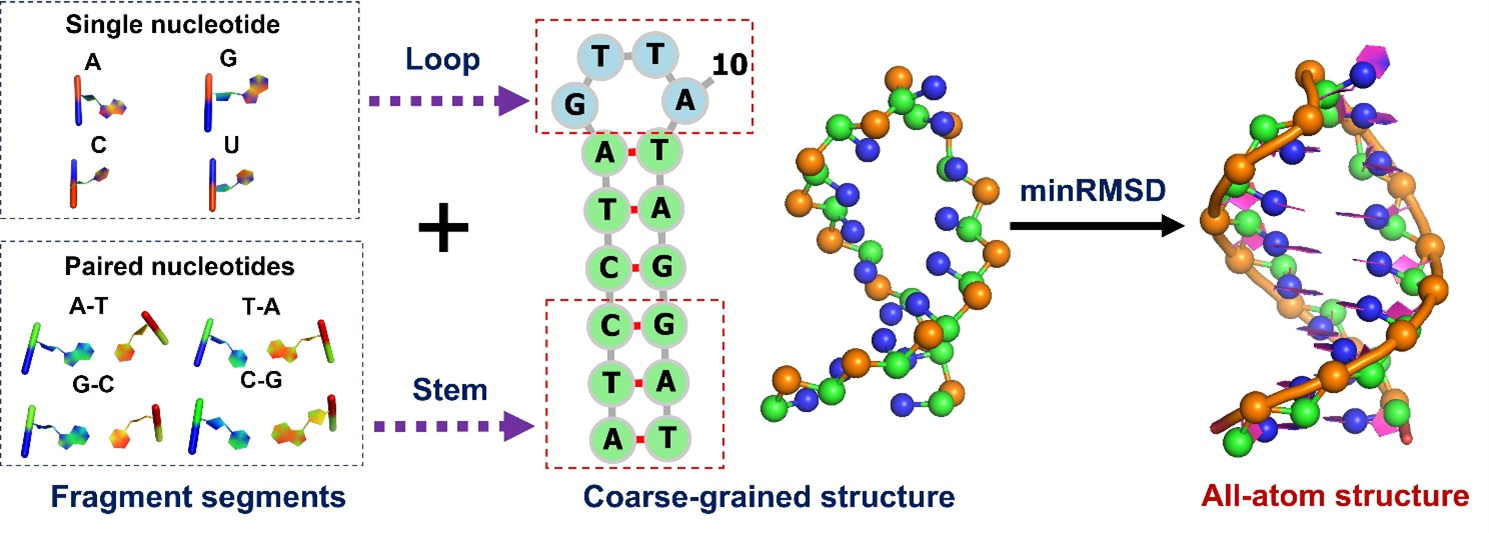

Supplement: S2 Fig — (TIF) [file pcbi.1013346.s002.tif]

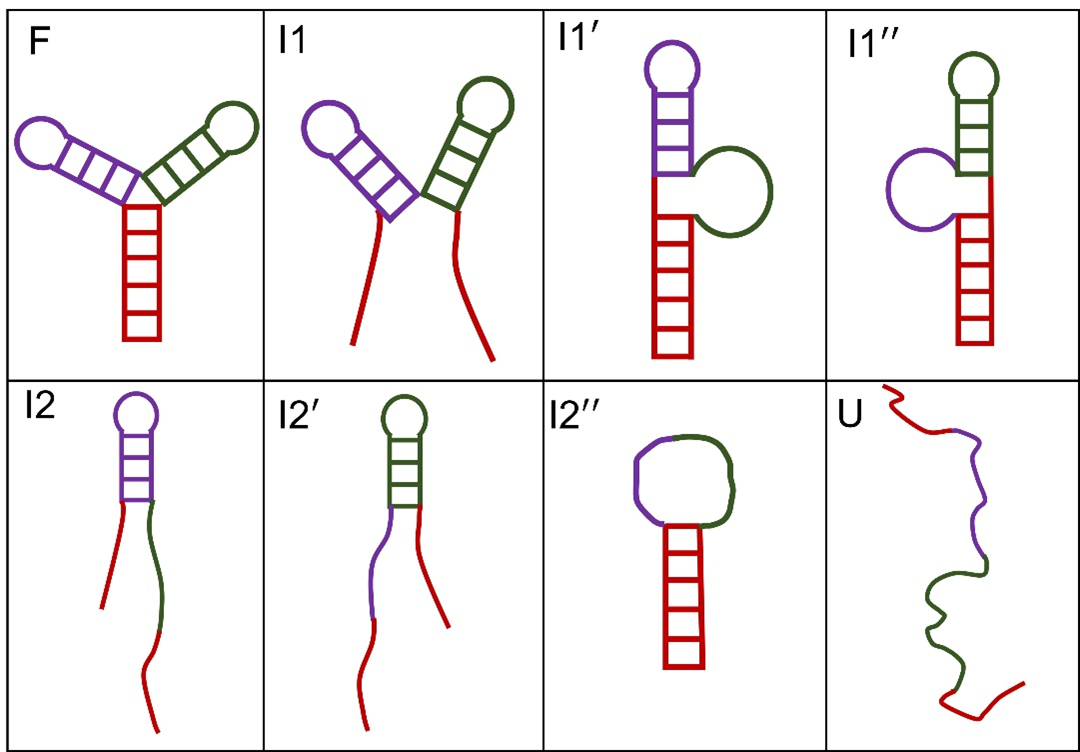

Supplement: S3 Fig — Here, F state: Stems 1, 2, and 3 retained, I1: Stem 1 resolved, I1′: Stem 3 resolved, I1′′: Stem 2 resolved, I2: Stems 1 and 3 resolved, I2′: Stems 1 and 2 resolved, I2′′: Stems 2 and 3 resolved, U: All stems resolved. (TIF) [file pcbi.1013346.s003.tif]

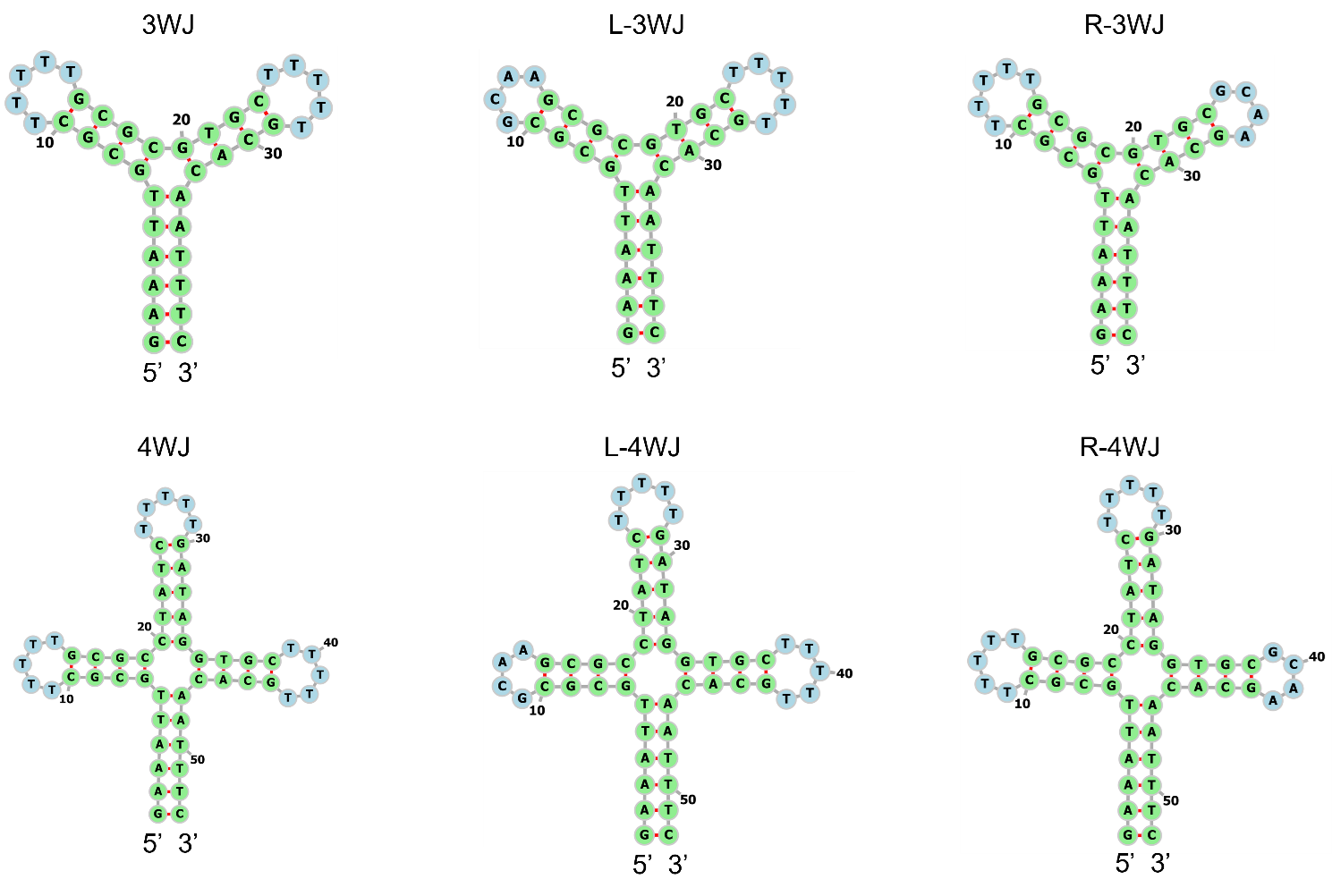

Supplement: S4 Fig — (TIF) [file pcbi.1013346.s004.tif]

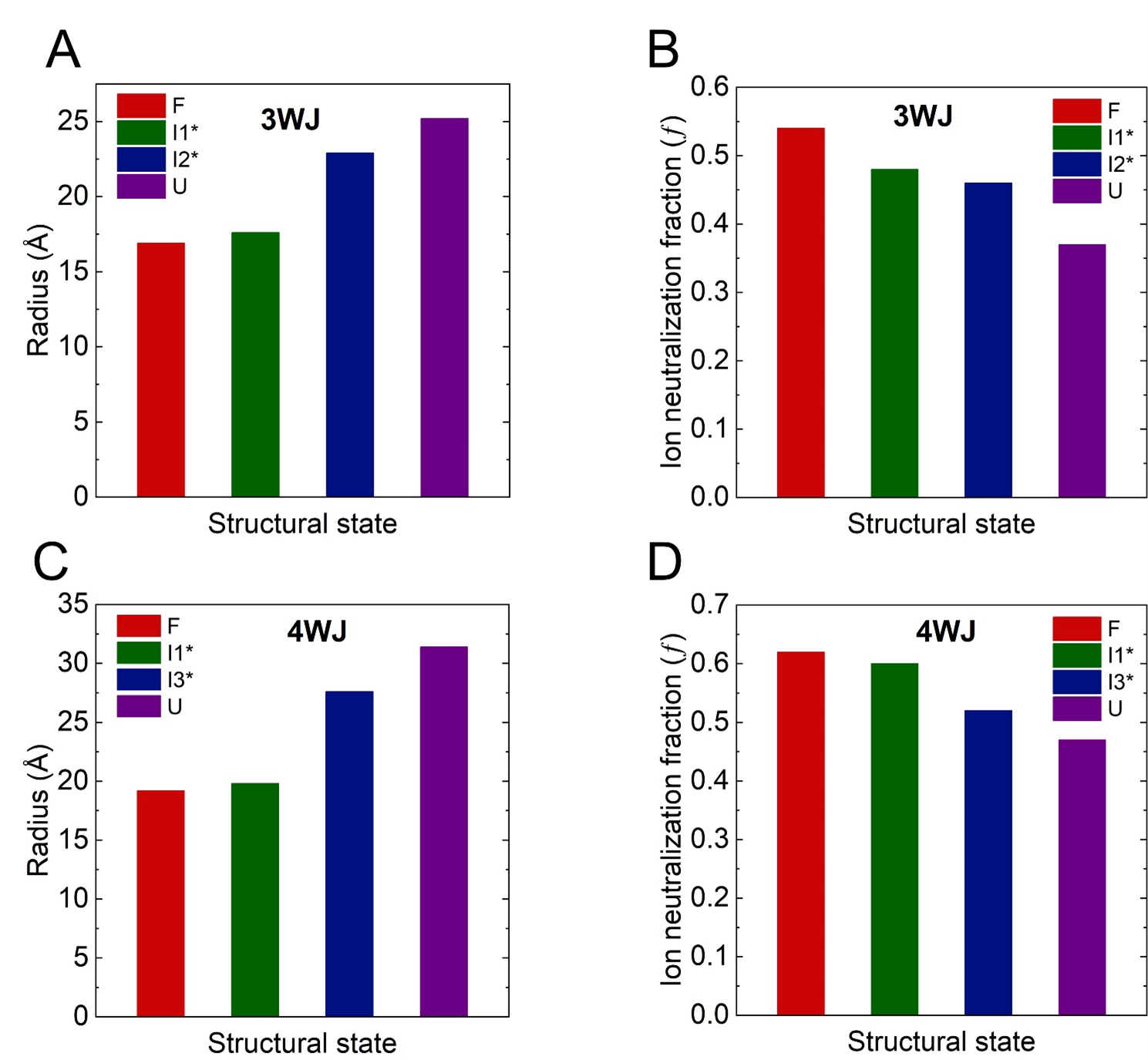

Supplement: S5 Fig — (A,B) The average Rg (A) and ion neutralization fraction (B) of folded state (F), intermediate states I1*/I2* (two stems retained/one stem retained), unfolded state (zero stem retained) for 3WJ. (C,D) The average Rg (A) and ion neutralization fraction (B) of folded state (F), intermediate states I1*/I3* (three stems retained/one stem retained), unfolded state (zero stem retained) for 4WJ. (TIF) [file pcbi.1013346.s005.tif]

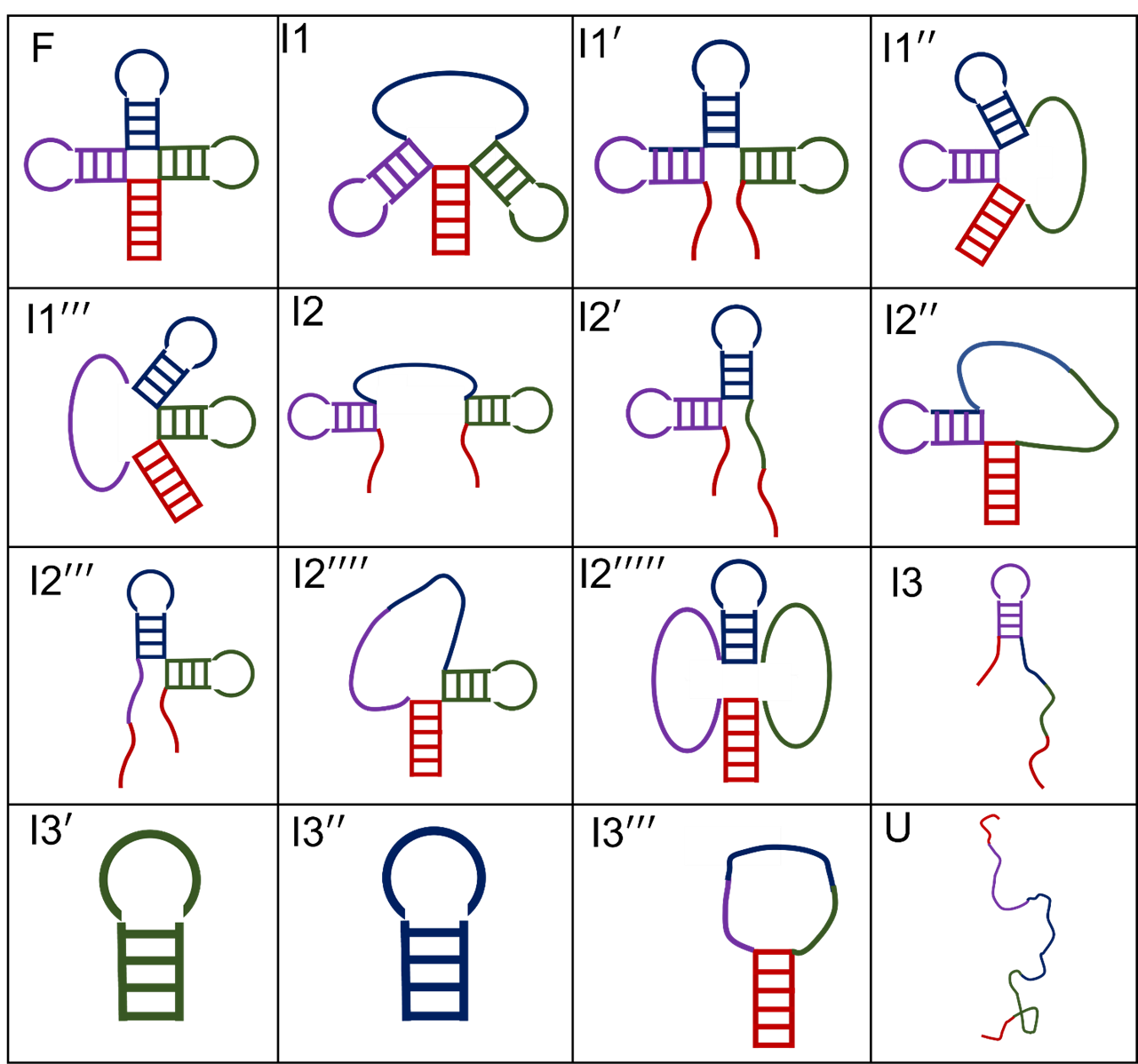

Supplement: S6 Fig — Here F: All stem retained, I1: Stem 3 resolved, I1′: Stem 1 resolved, I1′′: Stem 4 resolved, I1′′′: Stem 2 resolved, I2: Stems 1 and 3 resolved, I2′: Stems 1 and 4 resolved, I2′′: Stems 2 and 3 resolved, I2′′′: Stems 1 and 2 resolved, I2′′′′: Stems 2 and 3 resolved, I2′′′′′: Stems 2 and 4 resolved, I3: Stems 1,3, and 4 resolved, I3′: Stems 1, 2, and 3 resolved, I3′′: Stems 1, 2, and 4 resolved, I3′′′: Stems 2, 3, and 4 resolved, U: All stems resolved. (TIF) [file pcbi.1013346.s006.tif]
