# Supplementary material for: 3D structure and stability prediction of DNA with multi-way junctions in ionic solutions
Source: PLoS Comput Biol. 2025 Aug 18;21(8):e1013346. doi: 10.1371/journal.pcbi.1013346 (PMC12373291; doi:10.1371/journal.pcbi.1013346)
Supplement: S1 Text — (DOCX) [file pcbi.1013346.s013.docx]

**Supporting Information**

**3D structure and stability prediction of DNA with multi-way junctions in ionic solutions**

Xunxun Wang^1^, Ya-Zhou Shi^2,*^

*^1^Guizhou Key Laboratory of Microbio and Infectious Disease Prevention & Control, School of Biology and Engineering, Guizhou Medical University, Guiyang 550025, China*

*^2^Research Center of Nonlinear Science and School of Mathematics & Statistics, Wuhan Textile University, Wuhan 430073, China*

*To whom correspondence should be addressed: [yzshi@wtu.edu.cn](mailto:yzshi@wtu.edu.cn).

**The coarse-grained force filed of our present model**

The total potential energy $U$ for the present coarse-grained (CG) model is composed of eight distinct components, as outlined in [1].

$U=U_{b}+U_{a}+U_{d}+U_{bs}+U_{bp}+U_{exc}+U_{cs}+U_{el}$, (S1)

the first three terms represent the bonded energy contributions: $U_{b}$​ for the virtual bonded length, $U_{a}$ for the bonded angle, and $U_{d}$​ for the dihedral angle. These terms describe the connectivity and local geometry of the DNA chain. The expressions for $U_{b}$​, $U_{a}$​, and $U_{d}$​ are provided in [2].

$U_{b}=\sum_{bonds} K_{b}{(r-r_{0})}^{2}$; (S2)

$U_{a}=\sum_{angles} K_{\theta}{(\theta-\theta_{0})}^{2}$; (S3)

$U_{d}=\sum_{dihedrals} \left\{ K\left[ 1-\cos\left( -{}_{0} \right) \right]+\frac{1}{2}K\left[ 1-cos3 \left( -{}_{0} \right) \right] \right\}$, (S4)

the parameters $K_{b}$​, $K_{\theta}$​, and $K$​ represent the energy strengths, while $r_{0}$​, $\theta_{0}$, and $\varphi_{0}$ correspond to the equilibrium distances and angles for the virtual bonds, bond angles, and dihedral angles, respectively, at the energy minimum. To derive these bond energy parameters, we followed a systematic procedure. First, a statistical analysis of experimental DNA structures from the Protein Data Bank (PDB) was conducted to obtain the distance and angle distributions associated with the CG atoms. This dataset included 138 DNA structures, with ssDNA and dsDNA structures (see S1 Table). Next, we employed the functional forms outlined in Eqs. S2–S5 to fit the corresponding distance and angle distributions. From these fits, the bonded energy parameters were determined according to the following equation

$U\left( x \right)=-k_{B}T\ln\left[ P\left( x \right) \right]$, (S5)

where $k_{B}$​ denotes the Boltzmann constant, and $T$ represents the absolute temperature in Kelvin. The function $P\left( x \right)$ refers to the normalized distributions of the bonded distances and angles $x$. The model defines two sets of bond energies: Para_loop_ and Para_helix_. The Para_loop_ bond energy is used to model the folding of DNA treated as a free chain, derived from the single-stranded or loop regions (non-helix parts) in experimental structures. In contrast, the Para_helix_ bonded energy is employed during structure refinement. It is obtained from the stems (helix regions) in the experimental structures and is also used for the base-pairing nucleotides in the native-like structures predicted during the folding process. Further details can be found in Refs [1].

In Eq. S1, $U_{bs}$​ denotes the base-stacking interaction between two adjacent base pairs. The expression for $U_{bs}$ given by:

$U_{bs}=\frac{1}{2}$ $\underset{i,j}{\overset{N_{st}}{\sum}}\left| G_{i,i+1,j-1,j} \right|\left\{ \left[ 5\left( \frac{{}_{st}}{r_{i,i+1}} \right)^{12}-6\left( \frac{{}_{st}}{r_{i,i+1}} \right)^{10} \right]+\left[ 5\left( \frac{{}_{st}}{r_{j,j+1}} \right)^{12}-6\left( \frac{{}_{st}}{r_{j,j+1}} \right)^{10} \right] \right\}$, (S6)

here, ${}_{st}$​ represents the optimal distance between two neighboring bases in known helical structures. $G_{i,i+1,j-1,j}$​ denotes the base stacking energy strength, which was estimated by combining experimental

thermodynamic parameters and Monte Carlo simulations based on our previous model [2,3]:

$G_{i,i+1,j-1,j}=\Delta H-T\left( \Delta S-{\Delta S}_{C} \right).$ (S7)

In our CG model, $\Delta H$ and $\Delta S$ represent the DNA thermodynamic parameters obtained from experimental thermodynamic studies [4,5], while ${\Delta S}_{C}$ denotes the conformational entropy change, which is inherently accounted for in the Monte Carlo (MC) algorithm due to the formation of a base-pairing stack. The value of ${\Delta S}_{C}$​ was computed through MC simulations for the DNA helix, as illustrated in S1 Fig. During these simulations, the entire DNA molecule was kept fixed, except for the nucleotides indexed by $\leq i$ or $\geq j$, and the number of conformations $\Omega$, that satisfy the condition for base stacking between pairs ($i, j$) and ($i+1, j-1$) was counted, without imposing base-pairing and base-stacking constraints. The conformational entropy change for base stacking between pairs ($i, j$) and ($i+1, j-1$) was then calculated by:

${\Delta S}_{c}=k_{B}\ln\left( \Omega/{\Omega_{0}} \right)$, (S8)

where $k_{B}$​ represents the Boltzmann constant, and $\Omega_{0}$​ is the total number of conformations sampled during the simulation. ${\Delta S}_{C}$​ exhibits minimal variation across different base pair positions $i$. Therefore, for simplicity, an average value of -13.3 eu was adopted in the present model

The base-pairing potential is computed for all possible base pairs (G-C, G-U, and A-U), and is expressed as:

$U_{bp}=$ $\underset{i<j-3}{\overset{N_{bp}}{\sum}}\frac{{}_{bp}}{1+k_{NN}{(r_{N_{i}N_{j}}-r_{NN})}^{2}+k_{CN}\sum_{i(j)} \left( r_{C_{i}N_{j}}-r_{CN} \right)^{2}+k_{PN}\sum_{i(j)} \left( r_{P_{i}N_{j}}-r_{PN} \right)^{2}}$, (S9)

where ${}_{bp}$ is the interaction strength $\varepsilon_{AT}=$2*$\varepsilon_{GC}$/3. $r_{NN}$, $r_{CN}$, and $r_{PN}$ are three distances between the corresponding atoms of P, C and N in two paired nucleotides to describe the orientation of hydrogen-bonding interactions, and the values of them were obtained from the pairing bases in the PDB structures. Additionally, $k_{NN}$, $k_{CN}$ and $k_{PN}$ in Eq. S6 are the corresponding energy strength.

$U_{exc}$​ in Eq. S1 represents the repulsive volume interaction between CG beads, which is modeled using a purely repulsive Lennard-Jones potential. $U_{exc}=\sum_{i<j}^{N} \left\{ \begin{aligned} 4\varepsilon\left[ \left( \frac{\sigma_{0}}{r_{ij}} \right)^{12}-\left( \frac{\sigma_{0}}{r_{ij}} \right)^{6} \right], &if r_{ij}< \sigma_{0} \\ 0, &if r_{ij}\geq\sigma_{0} \end{aligned} \right.$, (S10)

here, *ε* = 0.26 kcal/mol represents the interaction strength, $\sigma_{0}$​ is the sum of the radii of beads $i$ and $j$, and $r_{ij}$ is the distance between beads *i* and *j*.

$U_{CS}$​ in Eq. S1 represents the coaxial-stacking interaction between two adjacent base pairs from two discontinuous stems [1,6].

$U_{cs}=\frac{1}{2}$ $\underset{i-j,k-l}{\overset{N_{cst}}{\sum}}\left| G_{i,k,l,j} \right|\left\{ \left[ 1-e^{-a\left( r_{ik}-r_{cs} \right)} \right]^{2}-\left[ 1-e^{-a\left( r_{jl}-r_{cs} \right)} \right]^{2}-2 \right\}$, (S11)

where $G_{i,k,l,j}$​ represents the base-stacking energy strength, approximating the base-stacking interaction between discontinuous stems and their nearest-neighbor base pairs. The distances $r_{ik}$ (or $r_{jl}$) correspond to the distances between the interfacing bases $i\left( j \right)$ and $k\left( l \right)$ of the two stems. The parameter $a$ describes the extent of the coaxial stacking distance, while $r_{cs}$ is the optimal distance between two coaxially stacked stems, derived from a statistical analysis of known structures in the PDB.

$U_{el}$​ in Eq. S1 represents the electrostatic interaction between phosphates, with their reduced charges determined by the counterion condensation model [7] and the tightly bound ion model [8-10]

$U_{el}=$ $\underset{i<j}{\overset{N}{\sum}}\frac{Q_{i}Q_{j}e^{2}}{4\pi\epsilon_{0}\epsilon r_{ij}}e^{-\frac{r_{ij}}{l_{D}}}$, (S12)

where $r_{ij}$​ represents the distance between the $i$-th and $j$-th phosphate beads, and $l_{D}$​ is the Debye length. The reduced charge on the $i$-th phosphate bead is

$Q_{i}=1-f_{i}$, (S13)

where $f_{i}$​ represents the ion neutralization fraction for the $i$-th phosphate bead. In addition to the assumption of a uniform distribution of binding ions along the DNA strand, $f_{i}$ depends on the DNA structure and incorporates both monovalent and divalent ions

$f_{i}=xf_{i}^{1}+\left( 1-x \right)f_{i}^{2}$, (S14)

where $f_{i}^{\nu} \left( \nu=1, 2 \right)$ represents the binding fraction of $\nu$-valent ions for the $i$-th phosphate bead. The terms $x$ and $\left( 1-x \right)$ denote the contribution fractions of monovalent and divalent ions, respectively. In Eq. S13, which is derived from the TBI model [8-10]. When using Na^+^ and Mg^2+^ to represent monovalent and divalent ions, $x$ can be expressed using an empirical formula [8-10].

$x=\frac{[\mathrm{Na}^{+}]}{[\mathrm{Na}^{+}]+\alpha[\mathrm{Mg}^{2+}]}$, (S15)

where $\alpha=\left( 8.1-64.8/N \right)\left( 5.2-\ln[\mathrm{Na}^{+}] \right)$, [Na^+^] and [Mg^2+^] represent the bulk concentrations of sodium and magnesium ions, respectively, and *N* is the length of the DNA chain [8-10]. For further details, please refer to Refs [8-11].

To achieve a more precise electrostatic potential for an DNA structure, $f_{i}^{\nu}$​ is defined as

$f_{i}^{\nu}=\frac{N\bar{f}_{i}^{\nu}}{\sum_{N} e^{-\beta{\nu\phi}_{i}}}e^{-\beta{\nu\phi}_{i}}$, (S16)

where $\bar{f}_{i}^{\nu}=1-(\frac{b}{\nu l_{B}})$​ represents the average neutralization fraction for the $i$-th phosphate bead [5]. In this expression, $b$ denotes the average charge spacing along the DNA backbone, and $l_{B}$​ is the Bjerrum length. The electrostatic potential $\phi_{i}$ for the $i$-th phosphate bead can be approximated as follows:

$\phi_{i}=$ $\underset{i\neq j}{\overset{N}{\sum}}\frac{l_{B}Q_{j}}{r_{ij}}e^{-\frac{r_{ij}}{l_{D}}}.$ (S17)

The reduced fraction $Q_{i}$​ based on the DNA structure is determined through an iterative procedure: (1) Start by calculating the neutralization fraction $f_{i}^{\nu}$ using Eq. S14, with the initial value ${f_{i}^{\nu}=\bar{f}}_{i}^{\nu}$; (2) Compute $Q_{i}$ using Eq. S13, then substitute $Q_{i}$ ​ into Eq. S17 to determine $\phi_{i}$; (3) Use Eq. S16 to calculate the fraction $f_{i}^{\nu}$ for the $\nu$-valent ion; (4) Repeat steps (1) through (3) until the value of $f_{i}^{\nu}$​ converges.

**Weighted histogram analysis method**

In our CG model, we employed the Weighted Histogram Analysis Method (WHAM) [12] to calculate the fractions of each DNA state at various temperatures, utilizing REMC trajectories. This approach enables the analysis of DNA thermal stability, including properties such as melting temperatures and thermally unfolding pathways. Specifically, the thermal stability of an DNA is predicted through the following four steps: First, we generate REMC trajectories for the DNA across a range of temperatures. Second, we discretize the relevant reaction coordinates-structural states ($S$) and energies ($E$)-into bins: $S_{j}$ ($j=1, 2,\ldots.,F$) and $E_{k}$ ($k=1, 2,\ldots,100$), where $S$ represents the structural states (e.g., folded, unfolded, and intermediate states) and $E$ represents the energies of conformations. Each pair of indices $(j,k)$ corresponds to a small state in the WHAM framework. For illustration, we use a DNA with three-way junctions as an example of an DNA structural state. The probability of small state $(j,k)$ at the *i-*th temperature, $p_{i,(j,k)}$​, is given by

$p_{i,(j,k)}=Z_{i}c_{i(j,k)}p_{(j,k)}^{^{\circ}}$, (S18)

where $p_{(j,k)}^{^{\circ}}$​ represents the unbiased probability of a small state $(j,k)$ at the temperature of interest, with the partition function $Z_{i}$ chosen such that the sum of all small states satisfies $\sum_{j,k} p_{i,\left( j,k \right)}=1$, ensuring normalization. The temperature-biasing factor $c_{i,(j,k)}$ is defined as $c_{i,(j,k)}=exp[-(\beta_{i}-\beta_{0})E_{k}]$, where $\beta_{i}$ is the inverse temperature at the *i-*th temperature, and $E_{k}$​ is the energy of conformation $k$. The unbiased probabilities $p_{(j,k)}^{^{\circ}}$​ for each small state are then determined by iterating the relevant equations until convergence is achieved.

$p_{(j,k)}^{^{\circ}}$ $=\frac{\sum_{i=1}^{M} n_{i, (j,k)}}{\sum_{i=1}^{M} N_{i}Z_{i}c_{i, (j,k)}}$, (S19)

$Z_{i}^{-1}=\sum_{j,k} c_{i, (j,k)}p_{(j,k)}^{^{\circ}}$. (S20)

where $M$ represents the number of replicas, with $M=10$ in our case. The quantity $n_{i,(j,k)}$​ denotes the count of small state $(j,k)$ at the *i-*th temperature, and $N_{i}$​ is the total number of conformations at the *i-*th temperature. Initially, the partition function $Z_{i}$​ for each temperature is set to 1. Finally, the fraction $f_{S_{j}}(T)$ of each structural state of DNA at temperature $T$ is calculated using the following equation

$f_{S_{j}}\left( T \right)=\sum_{k=1}^{100} p_{(j,k)}^{^{\circ}}$, (S21)

where $S_{j}$​ represents the different structural states of DNA, including folded (F), unfolded (U), and intermediate (I) states. The fraction $f_{S_{j}}(T)$ can be used to investigate the thermal stability and unfolding pathways of DNAs. Specifically, the melting temperature is determined by fitting the fractions of the folded state $f_{F}(T)$ ($F=S_{F}$) and the unfolded state $f_{U}(T)$ ($U=S_{1}$) to a two-state model.

$f_{F}\left( T \right)=\frac{1}{1+e^{(T-T_{m1})/dT_{1}}}$; (S22)

$f_{U}\left( T \right)=1-\frac{1}{1+e^{(T-T_{m2})/dT_{2}}}.$ (S23)

Here, $T_{m1}$ and $T_{m2}$​ denote the melting temperatures corresponding to the transitions from folded to intermediate (F$\to$I) and from intermediate to unfolded (I$\to$U), respectively. $dT_{1}$ and $dT_{2}$​ are the adjustable parameters associated with these transitions

**Reference**

1. Z.-C. Mu, Y.-L. Tan, B.-G. Zhang, J. Liu, Y.-Z. Shi, Ab initio predictions for 3D structure and stability of single-and double-stranded DNAs in ion solutions. *PLoS Comput. Biol* **18**, e1010501 (2022).

2. X. Wang, Y. L. Tan, S. Yu, Y. Z. Shi, Z. J. Tan, Predicting 3D structures and stabilities for complex RNA pseudoknots in ion solutions. *Biophys. J* **122**, 1503-1516 (2023).

3. Y. Z. Shi, F. H. Wang, Y. Y. Wu, Z. J. Tan, A coarse-grained model with implicit salt for RNAs: predicting 3D structure, stability and salt effect. *J. Chem. Phys* **141**, 105102 (2014).

4. J. SantaLucia, H. T. Allawi, P. A. Seneviratne, Improved nearest-neighbor parameters for predicting DNA duplex stability. *Biochemistry* **35**, 3555-3562 (1996).

5. J. SantaLucia Jr, D. Hicks, The thermodynamics of DNA structural motifs. *Annu. Rev. Biophys. Biomol. Struct.* **33**, 415-440 (2004).

6. Y. Z. Shi, L. Jin, C. J. Feng, Y. L. Tan, Z. J. Tan, Predicting 3D structure and stability of RNA pseudoknots in monovalent and divalent ion solutions. *PLoS Comput. Biol* **14**, e1006222 (2018).

7. G. S. Manning, The molecular theory of polyelectrolyte solutions with applications to the electrostatic properties of polynucleotides. *Q. Rev. Biophys* **11**, 179-246 (1978).

8. Z. J. Tan, S. J. Chen, Electrostatic correlations and fluctuations for ion binding to a finite length polyelectrolyte. *J. Chem. Phys* **122**, 044903 (2005).

9. Z. J. Tan, S. J. Chen, Electrostatic free energy landscapes for nucleic acid helix assembly. *Nucleic Acids Res* **34**, 6629-6639 (2006).

10. Z. J. Tan, S. J. Chen, Nucleic acid helix stability: effects of salt concentration, cation valence and size, and chain length. *Biophys. J* **90**, 1175-1190 (2006).

11. L. Jin, Y. Z. Shi, C. J. Feng, Y. L. Tan, Z. J. Tan, Modeling structure, stability, and flexibility of double-stranded RNAs in salt solutions. *Biophys. J* **115**, 1403-1416 (2018).

12. S. Kumar, J. M. Rosenberg, D. Bouzida, R. H. Swendsen, P. A. Kollman, The weighted histogram analysis method for free‐energy calculations on biomolecules. I. The method. *J. Comput. Chem* **13**, 1011-1021 (1992).
